# Genome-wide genotyping data renew knowledge on genetic diversity of a worldwide alfalfa collection and give insights on genetic control of phenology traits

**DOI:** 10.3389/fpls.2023.1196134

**Published:** 2023-07-05

**Authors:** Marie Pégard, Philippe Barre, Sabrina Delaunay, Fabien Surault, Djura Karagić, Dragan Milić, Miroslav Zorić, Tom Ruttink, Bernadette Julier

**Affiliations:** ^1^ INRAE P3F, Lusignan, France; ^2^ Login EKO doo, Bulevar Zorana Đinđića 125, Novi Beograd, Serbia; ^3^ International Maize and Wheat Improvement Center (CIMMYT), Nairobi, Kenya; ^4^ ILVO Plant Science Unit, Melle, Belgium

**Keywords:** alfalfa, genetic diversity, phenology, GWAS, genomic prediction

## Abstract

China’s and Europe’s dependence on imported protein is a threat to the food self-sufficiency of these regions. It could be solved by growing more legumes, including alfalfa that is the highest protein producer under temperate climate. To create productive and high-value varieties, the use of large genetic diversity combined with genomic evaluation could improve current breeding programs. To study alfalfa diversity, we have used a set of 395 alfalfa accessions (i.e. populations), mainly from Europe, North and South America and China, with fall dormancy ranging from 3 to 7 on a scale of 11. Five breeders provided materials (617 accessions) that were compared to the 400 accessions. All accessions were genotyped using Genotyping-by-Sequencing (GBS) to obtain SNP allele frequency. These genomic data were used to describe genetic diversity and identify genetic groups. The accessions were phenotyped for phenology traits (fall dormancy and flowering date) at two locations (Lusignan in France, Novi Sad in Serbia) from 2018 to 2021. The QTL were detected by a Multi-Locus Mixed Model (mlmm). Subsequently, the quality of the genomic prediction for each trait was assessed. Cross-validation was used to assess the quality of prediction by testing GBLUP, Bayesian Ridge Regression (BRR), and Bayesian Lasso methods. A genetic structure with seven groups was found. Most of these groups were related to the geographical origin of the accessions and showed that European and American material is genetically distinct from Chinese material. Several QTL associated with fall dormancy were found and most of these were linked to genes. In our study, the infinitesimal methods showed a higher prediction quality than the Bayesian Lasso, and the genomic prediction achieved high (>0.75) predicting abilities in some cases. Our results are encouraging for alfalfa breeding by showing that it is possible to achieve high genomic prediction quality.

## 1 Introduction

Alfalfa (*Medicago sativa*) is a major legume forage species grown worldwide. Its positive impact on protein autonomy through atmospheric nitrogen fixation and the environmental services it provides at the plot and rotation level have become increasingly important in recent years (Julier et al., 2017). This suggests that alfalfa should be grown on expanding areas (Poux and Aubert, 2018; Martin et al., 2020). Breeding is a lever to improved forage yield and quality as well as tolerance to biotic and abiotic stresses (Rubiales et al., 2021). Two aspects are critical: the use of genetic variation in which the selection is applied and the identification of the best genotypes, which will contribute to create the next generation.

From its origin in the Middle East, the domestication and breeding history of alfalfa in the Western world (Michaud et al., 1988; Lesins and Lesins, 2012) as well as in Asia (Basigalup et al., 2014) have previously been described (Small, 2011). Two main subspecies have been described: ssp *sativa* with purple flowers, a tap-root and coiled pods; and ssp *falcata* with yellow flowers, fasciculate roots and silk-shape pods. Even if the two subspecies can be intercrossed, the cultivated varieties mostly relate to the ssp *sativa* type with various levels of introgression with ssp *falcata*, which has conferred cold resistance and variegated flower colour. Studies based on molecular markers have revealed the genetic relationships among accessions (Li, 2013), the reduction of diversity in cultivated compared to wild populations (Muller et al., 2006) and described the genetic distance between Western and Asian accessions (Qiang et al., 2015). Markers have confirmed the huge within-accession diversity (Flajoulot et al., 2005) already observed with phenotypic traits (Julier et al., 2000). These studies have been conducted on small sets of diversity and/or with small sets of markers, and in several cases, the accessions were represented by a single individual. Thus, the description of alfalfa cultivated material is not yet optimal. At present, the use of within and among-accession diversity in breeding programs may be limited because of restricted access to this diversity and the fear that foreign/distant accessions do not comply with the breeders’ ideotype (Annicchiarico et al., 2015a). As with other species, a better knowledge of the genetic diversity of alfalfa could broaden the genetic basis of breeding programs and thus increase the potential for genetic gain.

Alfalfa breeding programs still rely on phenotypic selection in which the genetic value of a plant is evaluated directly on the plant (mass selection) or more accurately through its progeny under field or test conditions. In other species, numerous breeding programs have benefited from the advances in high-throughput genotyping technologies (Rasheed et al., 2017). With a large number of markers, it becomes possible to analyse and manage the genetic diversity, to identify markers involved in trait variation (Genome Wide Association Study) (Flint-Garcia et al., 2003) and to create genomic prediction equations to predict the genetic value with the marker information (Meuwissen et al., 2001). Known as genomic selection (GS), this last method has proved its efficiency in plant breeding (Crossa et al., 2017). A successful implementation of GS in breeders’ plant material requires considering certain parameters. Firstly, the linkage disequilibrium and the effective size of the population affect the number of markers needed to reach an accurate prediction: the number of required markers increases if linkage disequilibrium decays at shorter distance (Grattapaglia and Resende, 2011; Wientjes et al., 2013). Secondly, the composition of the population used to train (training population) the prediction model must be considered (Lorenz and Smith, 2015; Tayeh et al., 2015; Pégard et al., 2021). The training population must be representative of the selection candidates and several authors have studied the way in which it can be optimised (Rincent et al., 2012; Akdemir et al., 2015; Isidro et al., 2015). Thirdly, the trait genetic architecture will affect the prediction performance of the statistical methods (Wimmer et al., 2013), but this parameter is usually unknown and difficult to assess, requiring testing of multiple methods.

Genotyping alfalfa, an autotetraploid and allogamous species, has taken a leap forward with the use of Genotyping-by-Sequencing methodology (GBS), as described on heterozygous diploid species (Elshire et al., 2011). In the first attempts on alfalfa, marker calling was based on *de novo* assembly of reads without a reference genome (Li et al., 2014; Annicchiarico et al., 2015b; Biazzi et al., 2017) or on a mapping of the reads on the reference genome sequence of the related model species *Medicago truncatula* (Julier et al., 2018). With the recent release of tetraploid alfalfa reference genome sequences (Carrère et al., 2020; Chen et al., 2020; Shen et al., 2020; Long et al., 2022), more reads are expected to be mapped and the markers are physically positioned on the alfalfa genome. This GBS methodology offers a high throughput genotyping tool that is convenient for most of the genetic studies, at the individual level with the allele dosage determination as well as the population level with the allele frequency determination (Julier et al., 2018).

Linkage disequilibrium decays at short distance in allogamous species (Flint-Garcia et al., 2003) and this also applies to alfalfa (Herrmann et al., 2010). The candidate gene approach is appropriate for association mapping (Herrmann et al., 2010), but requires previous knowledge of relevant candidate genes. Conversely, performing a genome-wide association study (GWAS) with reduced representation libraries such as GBS, requires sequencing at many loci that are evenly spread across the genome and at high density. Such QTL have been obtained on diploid alfalfa (Sakiroglu and Brummer, 2017) or cultivated tetraploid alfalfa (Biazzi et al., 2017). Genomic prediction (Meuwissen et al., 2001) has been tested on alfalfa (Annicchiarico et al., 2015b; Li et al., 2015b; Biazzi et al., 2017; Medina et al., 2021; Andrade et al., 2022), showing promising predicting ability around 30% for forage yield and quality traits. Higher predicting ability could probably be obtained by using a larger population size and/or a lower percentage of missing data in the genotyping dataset and/or more markers at more loci.

In alfalfa, as in other species, phenology traits are the major drivers of climate adaptation. Flowers are formed at the leaf axillaries (Teuber and Brick, 1988) and do not hamper stem elongation. In contrast with most cultivated species, flowering date is not a component of forage yield in alfalfa, and it is not even scored by breeders. However, the beginning of flowering stage is used as an indicator for the cutting date since it indicates a good compromise between forage yield, quality and persistence. On the other hand, fall dormancy, defined as the reduction of growth in fall in response to short day length (Blondon et al., 1967), is a component of fall and spring yield. It is also a main, but not unique, component of winter frost tolerance (Teuber et al., 1998; Brummer et al., 2000; Willame et al., 2002; UPOV, 2005). Each breeding program is usually conducted within a restricted fall dormancy range to release varieties targeting a specific climate.

In this study, we gathered ‘cultivated material’, comprising old and recent cultivated accessions mainly from Europe, North and South America, and China, and further extended the genetic diversity with breeding material of five major European breeders. From this material, we assessed whether a genetic structure has been created by preferential crosses between materials of specific fall dormancy groups. We studied how diversified breeding material of the five European breeders is, compared to the diversity found in the cultivated material. With the cultivated material, we assessed if a GWAS approach can detect QTL for phenology traits and if the genomic prediction models allow to predict phenology with a good accuracy. In this study, GWAS and GP analyses were conducted at accession (*i.e.* population) level, with SNP frequencies as genotyping data.

## 2 Material and method

### 2.1 Plant material

We used 400 cultivated accessions (hereafter named as ‘cultivated material’) comprised of 378 cultivars and 22 landraces whose fall dormancy score mainly ranged between 3 and 7. Their origin, based on the place they have been collected (landraces) or initially selected and registered (cultivars) was Europe (318 accessions), North America (45 accessions), South America (16 accessions), China (17 accessions), Middle East (3 accessions) and Japan (1 accession). In addition, 617 accessions (hereafter named as ‘breeding material’) representing advanced breeding material obtained by five European breeders were included: 144 accessions from breeder A, 62 accessions from breeder B, 101 accessions from breeder C, 189 accessions from breeder D and 121 accessions from breeder E. Each breeder chose their material for this study but did not mention if it was used in or representative of the breeding program of the company or institution. All the 1017 accessions were genotyped and used for genetic structure study while only the 400 ‘cultivated material’ accessions were phenotyped for phenology and used for GWAS and GP studies. The origin of the material used is available in the data repository (see the Data Availability Statement section).

### 2.2 Genotyping

The methodology used for the DNA extraction, the optimization of the GBS methodology and GBS sequencing has previously been reported in Julier et al., 2021. To summarize, each accession was represented by 100 plants, the DNA extraction was performed from a pool of 100 leaflets, each taken on a plant. This protocol has previously been shown to be reliable to estimate the allele frequency of an accession (Julier et al., 2018). The double-digest GBS on alfalfa was conducted with the enzymes PstI-MseI to obtain a sufficient number of loci, while reducing the number of missing values and considering the number of reads per accession.

### 2.3 Trimming and SNP calling

**Figure 1:**
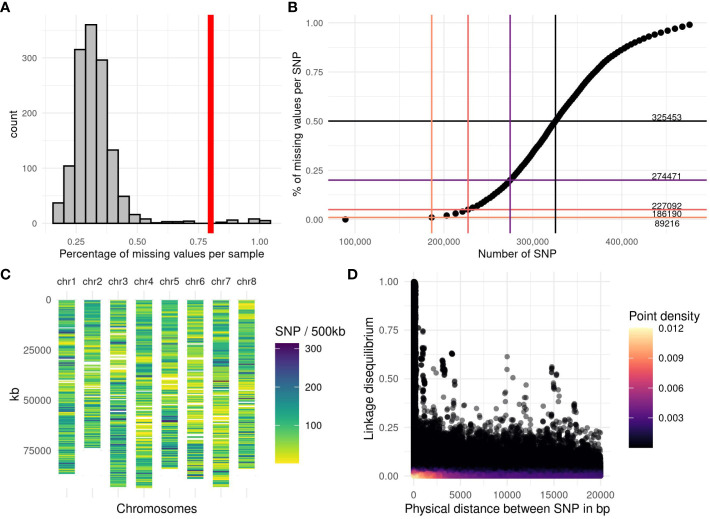
Genotyping quality and linkage disequilibrium observed among the accessions. **(A)** Histogram of the percentage of missing values per accession, the vertical red line represents the applied threshold of 80% of missing values per accession. **(B)** Number of SNPs available depending on the percentage of missing values allowed per SNP. The horizontal lines are the thresholds (1%, 5%, 20% and 50%), the vertical bars represent the number of SNPs obtained with the corresponding threshold. The number in the right part of the graph indicates the number of SNPs from 0% of missing value to 50% of missing values. **(C)** SNP density per chromosome along the genome, estimated by the number of markers in a window of 500 kb on the 227 092 SNPs obtained with a threshold of 5% missing value per SNP. **(D)** Linkage disequilibrium (LD) between the 227 092 SNPs, estimated with a squared partial correlation. The LD was plotted for SNP distances of less than 20 000 bp. The purple color scale represents the point density, black for a low density and yellow for the highest density.

The reads were preprocessed with the GBprocesS bioinformatics pipeline (Schaumont, 2020). This pipeline includes several steps: demultiplexing, trimming of barcodes and restriction enzyme cutsite remnants, merging of forward and reverse reads, removal of reads with low quality base-calling and internal restriction sites. Subsequently, the reads were mapped onto the reference sequence (Chen et al., 2020) by using the BWA software with the BWA-MEM algorithm and default options. We performed a test on a batch of samples to compare the number of SNPs when the reads were mapped on each of the four homologous chromosomes of the reference genome. The haploid copy of the genome giving the highest number of SNPs was chosen (number 2) as the reference to map the reads for the rest of the accessions. We used SMAP *delineate* (Schaumont et al., 2022) to analyse stacks of GBS reads mapped onto the reference sequence, and found 31 743 loci. A custom pipeline was used to perform the genotype calling. First, for each accession, the number of reads per position and per nucleotide (A, T, G, C) was extracted with the software bamreadcount (Khanna et al., 2022). Per accession, a threshold was applied to keep only the positions with at least 10 reads and at most 1200 reads. A list containing all the positions found across accessions was established which included 22 192 769 positions. For each position, the allele frequency of each nucleotide was calculated as the number of reads for the targeted nucleotide divided by the total number of reads at this position. Two stages of position selection were then carried out to retain the positions with a minor allele frequency greater than or equal to 1% and two alleles, leading to 1 194 485 positions. After this step, each accession was genotyped for the remaining positions by calculating the allele frequency of the alternative allele. In a third selection, we retained 631 816 positions with a minor allele frequency per accession between 5% and 50% in at least 10 accessions. Five accessions with more than 80% of missing data were excluded from the analysis (**Figure 1A**).

To allow robust analyses, it is necessary to filter out markers with too many missing data. When the percentage of missing data per SNP was plotted against the number of markers, we obtained a sigmoid curve (**Figure 1B**) on which we have represented different thresholds and the number of markers retained. By applying the thresholds of 0%, 1%, 5%, 20% and 50% missing data, 89 216, 186 190, 227 092, 274 471 and 325 453 SNPs were retained, respectively. In this study, we applied the filter of 5% maximum missing data per position (227 092 SNPs). We calculated the number of markers over a distance of 500 kb. This density of markers is variable along and between the chromosomes (**Figure 1C**) but the whole genome is covered, except for two zones which are certainly centromeres.

### 2.4 Population structure

A subset of 89 216 SNPs without missing values was used for a genetic structure analysis. Linkage disequilibrium decay was calculated based on the squared partial correlation between pairs of SNPs (Lin et al., 2012; Mangin et al., 2012). Genetic groups among the populations were identified with the Discriminant Analysis of Principal Components (DAPC) method (Pritchard et al., 2000; Jombart et al., 2010; Grünwald and Goss, 2011) implemented in the R package adegenet (Jombart, 2008; Jombart and Ahmed, 2011). The genetic groups were identified by using k-means, a clustering algorithm that found a given number (k) of groups by maximizing the variation between groups. The optimal number of groups was the one that provided the lowest Bayesian Information Criterion (BIC). We then ran a Principal Component Analysis (PCA) with the R package ‘FactoMineR’ (Lê et al., 2008) to analyze the diversity among accessions without prior hypothesis and their inclusion to each group was illustrated. The same R package (‘FactoMineR’) was used to project the breeders’ accessions (as supplementary individuals) into the PCA of the cultivated material. To illustrate the relationship with the genetic structure found with the SNPs and the fall dormancy, a PCA was performed on phenotypes (see next section) and the accessions were colored by group as found by the clustering analysis. We also used the results of the PCA based on the genomic information and colored the accessions depending on the phenotype for each trait. The differentiation between the groups was assessed through the F_ST_ value following the methods of the R package StAMPP (Pembleton et al., 2013) for polyploid species. The group with only two accessions was ignored for the F_ST_ estimation.

### 2.5 Phenotyping

All the “cultivated material” plant material was established in two locations for phenotyping, the first one in France at the research unit (URP3F) of INRAE (Lusignan: 46° 23’ 60’’ N, 0° 4’ 48’’ E) and the second one in Serbia at the research unit of IFVCNS (Novi Sad: 45° 15’ 0’’ N, 19° 51’ 0’’ E). In Lusignan, the trial was sown on the 10^th^ of May 2018. The trial was damaged by a storm on the 26^th^ of May 2018 and a new trial was sown on the 23^rd^ of August 2018, but some of the cultivated material did not have enough seeds, so only 387 among the original 400 accessions were established. In Novi Sad, the trial was sown on the 21^st^ of May 2018. The trials were composed of 440 plots, 44 columns and 10 rows, in an augmented block design with four incomplete blocks (Federer and Raghavarao, 1975; Lin and Poushinsky, 1985). The **Table S1** summarises the dimension and the technical elements of the trials. Five accessions were repeated six times and distributed in the four blocks, 15 other accessions were repeated twice in the trials of Lusignan-May 2018 and Novi Sad, but in Lusignan-August 2018, 28 accessions were repeated twice. The other accessions were present only once and randomly distributed within and between the blocks. Measurements and scorings were performed in each trial in 2018, 2019 and 2020. The trial installed in Lusignan was evaluated during an extra year in 2021. On the trial established in May 2018 in Lusignan, the number of surviving plants in each plot was enough to record the flowering date in summer 2018. The date of flowering (FD.L) was then converted into a degree.day sum, by adding up the degrees Celsius above zero between the date of sowing and the date of flowering, using the mean daily temperatures at the location and in the year of the trial. The assessment of fall dormancy was carried out by measuring several traits on the trials established in August 2018 in Lusignan (.L) and May 2018 in Novi Sad (.N): plant height (PH19.L, PH20.L, PH21.L, PH19.N, PH20.N) before the last cut in fall 2019 and 2020 and dry matter yield (F-DMY19.L, F-DMY21.L, F-DMY19.N, F-DMY20.N) at the last cut in fall 2019 and 2020. A fall dry matter yield combined over all the years and locations (F-DMY) was estimated with a mixed model to remove the year and the location effects. Fall plant height measurements were made with an electronic ruler when heights were less than 35 cm and with a conventional ruler when heights were greater than 35 cm. Three heights per plot were measured, randomly in the high plant density plots and on the most developed plants in the degraded plots. All measurement and cutting dates are available in **Table 1**. In Lusignan, plant height was measured several times between the last two cuttings in the fall 2019 and 2021, and the stem elongation speed (SE19.L, SE21.L, in cm/degree.day) was obtained by using the slope of the regression between the height and the date of measurement expressed in degrees.days above 0°C. Finally, fall dormancy was visually scored on the 29^th^ of October 2019, on a 1-11 scale based on regrowth height (D19.L). Due to a very dry fall in Lusignan in 2020, fall regrowth was not sufficient for a cutting and an estimation of dry matter yield.

**Table 1:** List of measurement dates of each trait and the prior cutting date in DD.MM.YYYY format.

Trait	Measurement date	Date of prior cut
*D19.L*	29.10.2019	17.09.2019
*F-DMY19.L*	19.11.2019	17.09.2019
*F-DMY21.L*	01.09.2021	28.07.2021
*F-DMY19.N*	16.10.2019	22.08.2019
*F-DMY20.N*	22.10.2020	11.08.2020
*PH19.L*	29.10.2019	17.09.2019
*PH20.L*	16.10.2020	28.07.2020
*PH21.L*	09.11.2021	28.07.2021
*PH19.N*	16.10.2019	22.08.2019
*PH20.N*	22.10.2020	11.08.2020
*SE19.L*	07.10.2019-14.10.2019-21.10.2019-29.10.2019-13.11.2019	17.09.2019
*SE21.L*	13.09.2021-22.09.2021-18.10.2021-09.11.2021	28.07.2021

Flowering date (FD), Dormancy (D), Fall Dry Matter Yield (F-DMY), plant height (PH), Speed of elongation (SE) for two years: 2019 (X19.X) and 2020 (X20.X) in two locations: Lusignan (.L) in France and Novi Sad (.N) in Serbia.

### 2.6 Phenotypic adjustment and genetic parameter estimation

All traits were independently adjusted to field micro-environmental heterogeneity with the breedR package (Muñoz and Sanchez, 2020). Within trials, to capture the spatial heterogeneity at the plot level, a random effect was fitted thanks to the use of the tensor product of two B-splines bases with a covariance structure for the random knot effects (RKE) to account for spatial variability along the rows and the columns of the field design (Cantet et al., 2005; Cappa and Cantet, 2007; Robbins et al., 2012; Cappa et al., 2015). We used a genomic based mixed model for each year and each location. The genomic estimated breeding values (GEBV) for each trait were estimated with the best linear unbiased prediction based model (GBLUP) (Whittaker, 2000; Meuwissen et al., 2001):


(1) y=μ+Zu+Ws+ ϵ

where y was the raw phenotypes, μ the global mean, u the vector of random additive effects following $\mathbb{N}\left(0,G\sigma_{a}^{2}\right)$ with $\sigma_{a}^{2}$ the additive variance and G the genomic relationship matrix between accessions, s was the vector of random spatial effects containing the parameters of the B-splines tensor product following $\mathbb{N}\left(0,S\sigma_{s}^{2}\right)$ with $\sigma_{s}^{2}$ the variance of the RKE for rows and columns and S the covariance structure in two dimensions, ϵ the vector of residual effects following $\mathbb{N}\left(0,I\sigma_{e}^{2}\right)$ with $\sigma_{e}^{2}$ the residual variance. The design matrix Z and W are identity matrices connecting the plots to the random effects. The method used to obtain the genomic relationship matrix *G* is explained in the next section. B-splines were anchored at a given number of knots for rows and columns, a high number of knots smooths out the surfaces. breedR optimized the knot numbers by an automated grid search based on the Akaike information criterion (Akaike, 1974). The micro-environmental plot effect was subtracted from the observed phenotype to obtain a spatially adjusted phenotype. For the repeated accessions, we calculated an accession mean of the spatially adjusted phenotypes for each trait.

This model was used to estimate the narrow sense heritability of the trait. To avoid inflated heritability (Heckerman et al., 2016), the variance explained by the spatial effect is integrated in the heritability formula:


(2) $$h^{2}=\;\frac{VarG}{VarG+VarE+VarR}$$

With VarG the additive variance, VarE the micro-environmental plot variance and VarR the residual. We used a multi-trait model on adjusted phenotypes coupled with information from relatedness between individuals based on genomic information (Calus and Veerkamp, 2011) to extract the genetic correlation between traits and compare the genetic correlation with the phenotypic correlation calculated from the Pearson’s correlation on adjusted phenotypes.

### 2.7 Relationship matrix estimation

The genomic relationship matrix (G) was based on (VanRaden, 2008), adapted to use allele frequencies (continuous values from 0 to 1) instead of allele dosage (Ashraf et al., 2014). The genotyping matrix (M) was normalized by the minimum allele frequency (P) to obtain the normalized genotyping matrix (Z) used to compute G , as follows:


(3) $$G=\frac{ZZ^{\prime }}{\frac{1}{n}\sum_{j=1}^{m}p_{j}(1-p_{j})}$$

The denominator is a scaling parameter, corresponding to the sum of the expected SNP variance across genotypes (Ashraf et al., 2014), where m represents the number of markers, $p_{j}$ equals the frequency of the j ^th^ marker, and n represents a scaling number to obtain a diagonal mean equal to 1. This has been recommended in previous studies on polyploid species (Ashraf et al., 2014; Cericola et al., 2018), with =16 , the diagonal mean was close to 1.

### 2.8 GWAS

The GWAS analyses were performed with the MLMM method (Segura et al., 2012), while taking into account the genetic structure of the “cultivated material” with the genomic relationship matrix. The MLMM method uses a stepwise mixed-model regression approach with forward inclusion of the SNP as co-factors and a backward elimination. The variance components of the model are re-estimated at each step. This method is known to increase the detection power while decreasing the false detection rate. The maximum number of steps was limited to ten. The best step is selected with an adjusted (0.05/number of GBS loci) multiple Bonferroni criterion (mBonf). The percentage of phenotypic variation explained by each QTL was obtained by subtracting the R² of a linear model with all the QTL as fixed effects and the genomic relationship matrix (G) as random effect to the R² of the same model but without the focused QTL.

Genes located within 2500 bp flanking each QTL were determined using the Genome Browser (https://bbric-pipelines.toulouse.inra.fr/myGenomeBrowser?portalname=MSAT_XinJiangDaYe&owner=sebastien.carrere@inrae.fr&key=PyG9k9tK). Three sources of gene annotation were available, one from the reference genome used for the genotype calling (Chen et al., 2020), one from a partly assembled European genome (Carrère et al., 2020) and one from the genome of the model legume species *Medicago truncatula* (Pecrix et al., 2018).

### 2.9 Genomic prediction

#### 2.9.1 Test of the size and the genetic composition of the training population

To assess the potential of genomic prediction, we used the predicting ability calculated by the correlation between the phenotype and the value predicted in the validation population. The validation population represents a portion of the complete dataset on which the phenotypes have been masked and only the genotype information is available. Here, two cases were considered. In a first case, 100 accessions were randomly taken to form the validation population, in a way that each group (see paragraph Genetic structure) is represented according to its size. We randomly sampled the remaining accessions to test the effect of the size of the training population. Nine sample sizes were tested: ranging from 10% of the remaining accessions (29 accessions) to 90% (270 accessions), ten iterations were performed for each sample size. In order to assess the effect of the training population composition on the quality of the predicting ability, a second case was studied. The validation population was composed of fifteen accessions from one group only and the training population of 210 accessions was randomly taken from the remaining groups. Ten repetitions were performed. Groups with less than fifteen accessions were excluded. For each repetition, the validation population was sampled from within a group and was predicted using two different training populations. To test the predicting ability across groups, the first training population excluded the other accessions belonging to the same group as the validation population. The second training population was a random sample of all groups. We ensured that the potential confusion between the effect of the composition of the training population and the effect of the sample size was avoided by using the same sampling but two different training populations.

#### 2.9.2 Test of statistical models

First, the best linear unbiased prediction based on genomic information (GBLUP) (Whittaker, 2000; Meuwissen et al., 2001) was used to predict the genomic estimated breeding values with all the SNP used to compute the genomic relationship matrix matrix (G). The R package breedR was used.

Then, we used two Bayesian methods, the Bayesian Ridge Regression (BRR) (Pérez et al., 2010) and the Bayesian Lasso (lasso) (Tibshirani, 1996; Tibshirani et al., 2012), with the R package glmnet (Friedman et al., 2010; Simon et al., 2011). The BRR and the GBLUP method mimic an infinitesimal genetic architecture. The Bayesian Lasso method selects features (here SNPs) depending on their importance and uses them as a predictor.

We compared the different models in the situation of the first case scenario with a validation population of 100 accessions randomly selected and a training population of 270 randomly selected accessions (90%).

## 3 Results

### 3.1 Genotyping

The GBS pipeline optimization, as performed in this study, led to a genotyping dataset with little missing values among the accessions. Only five accessions out of 1017 were lost due to a poor sequencing depth (**Figure 1A**). We chose to keep SNPs with a maximum of 5% missing values per SNP (**Figure 1B**), the missing values were imputed with the minor allele frequency, and it represented less than 1% of all the genomic data. We chose this low threshold to avoid adding bias in the QTL detection and the genomic prediction. GBS-tagged loci were spread fairly evenly throughout the genome, with at least 1 polymorphic SNP marker every 500 kb (**Figure 1C**). Linkage disequilibrium (LD), estimated with a squared partial correlation, dropped abruptly after 1000 bp, most SNP pairs presented a LD close to zero as shown by the yellow colour representing a high density (**Figure 1D**). Indeed, after a distance of 100 kb, on the 810 557 188 total pairs of SNP, only 872 SNP pairs showed a partial correlation >0.25; and 75 SNP pairs had a partial correlation >0.5. The longest distance between two SNPs were 89 Mb and 91 Mb, with a partial correlation of 0.5 and 0.25, respectively. Further examination revealed that this long-range LD is mainly due to a few pairs of SNP that have high LD with several flanking SNPs located in another region of the chromosome, suggesting a local problem in assembly or a wrong mapping.

### 3.2 Population structure

**Figure 2:**
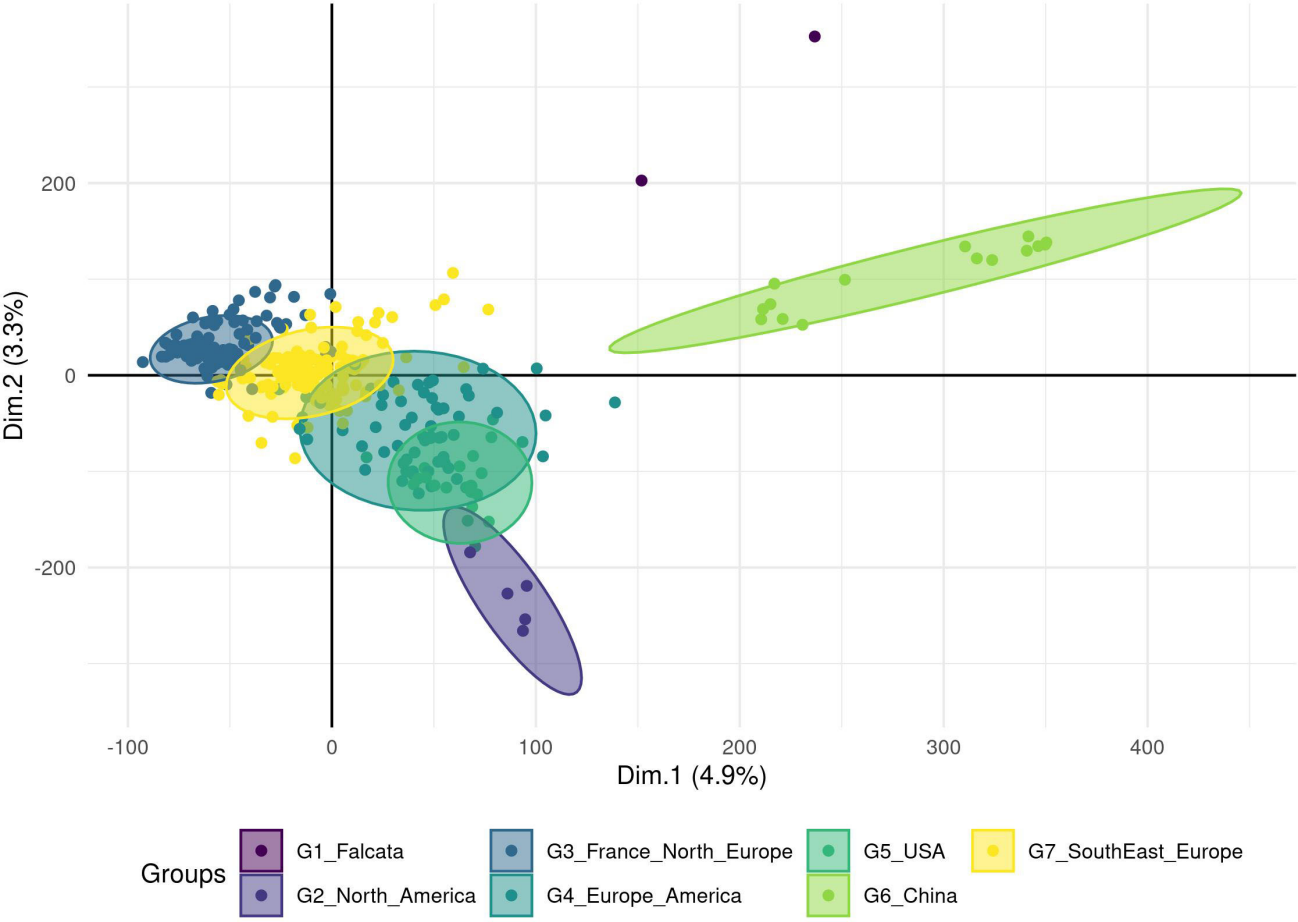
Clustering of the cultivated material based on a Principal Component Analysis performed on a genotyping dataset of 89 216 SNPs without missing data. The analysis was done on 395 accessions, and a Discriminant Analysis of Principal Components (DAPC) analysis revealed seven groups (ellipses) linked to the geographic region of the origin or registration.

The number of principal components (PC) required to explain 90% of the genetic variation was 300 (**Figure S1A**). The optimal number of groups was the one that provided the lowest Bayesian Information Criterion (BIC), here seven (**Figure S1B**). On the PCA with the accessions coloured according to the seven DAPC groups (**Figure 2**), two groups were clearly separated from the others: group 6 with 15 Chinese accessions and group 1 with two accessions (an Italian variety that includes a *falcata* parent and a Hungarian variety). The five other groups showed a genetic continuum but each group can be related to the geographic origin of the accessions: group 3 with 139 accessions of European origin (France and Northern Europe), group 7 with 151 accessions mostly of European origin (Southern and Eastern Europe), group 4 with 61 accessions of Europe, North and South America, group 5 with 21 USA and 1 Chinese accessions and group 2 with 5 North American accessions. The European accessions as well as the American accessions were thus split into several groups. The group 4 probably illustrates the multiple origins of some varieties selected in the two continents.

Finally, the European and the North American accessions displayed little overlap, but South-American accessions interestingly overlapped with these two groups. The three accessions from the Middle East were close to the American accessions and the Japanese accession was closer to the European-American groups than to the Chinese group. All Chinese varieties resided in the Chinese group except one variety that grouped into group 5, probably revealing a selection based on American material. Accessions from China seemed to be different from *falcata* material (group 1), even if the latter was represented by only two accessions in this study. The distance between the group 6 with Chinese accessions and the overlapping groups 3, 4, 5, 7 containing Western accessions suggested unconnected breeding programs. The F_ST_ value between the groups were low with an average value of 0.01 and a range between 0.001 and 0.026 (**Table 2**). Groups 6 and 2 were the most distinct, with the highest F_ST_ value (0.026).

**Table 2:** Genetic distinction (F_ST_) between the groups found by the DAPC method.

Groups	1	2	3	4	5	6
1					
2						
3		0.017				
4		0.013	0.003			
5		0.004	0.006	0.002		
6		0.026	0.019	0.012	0.015	
7		0.016	0.001	0.001	0.005	0.015

**Figure 3:**
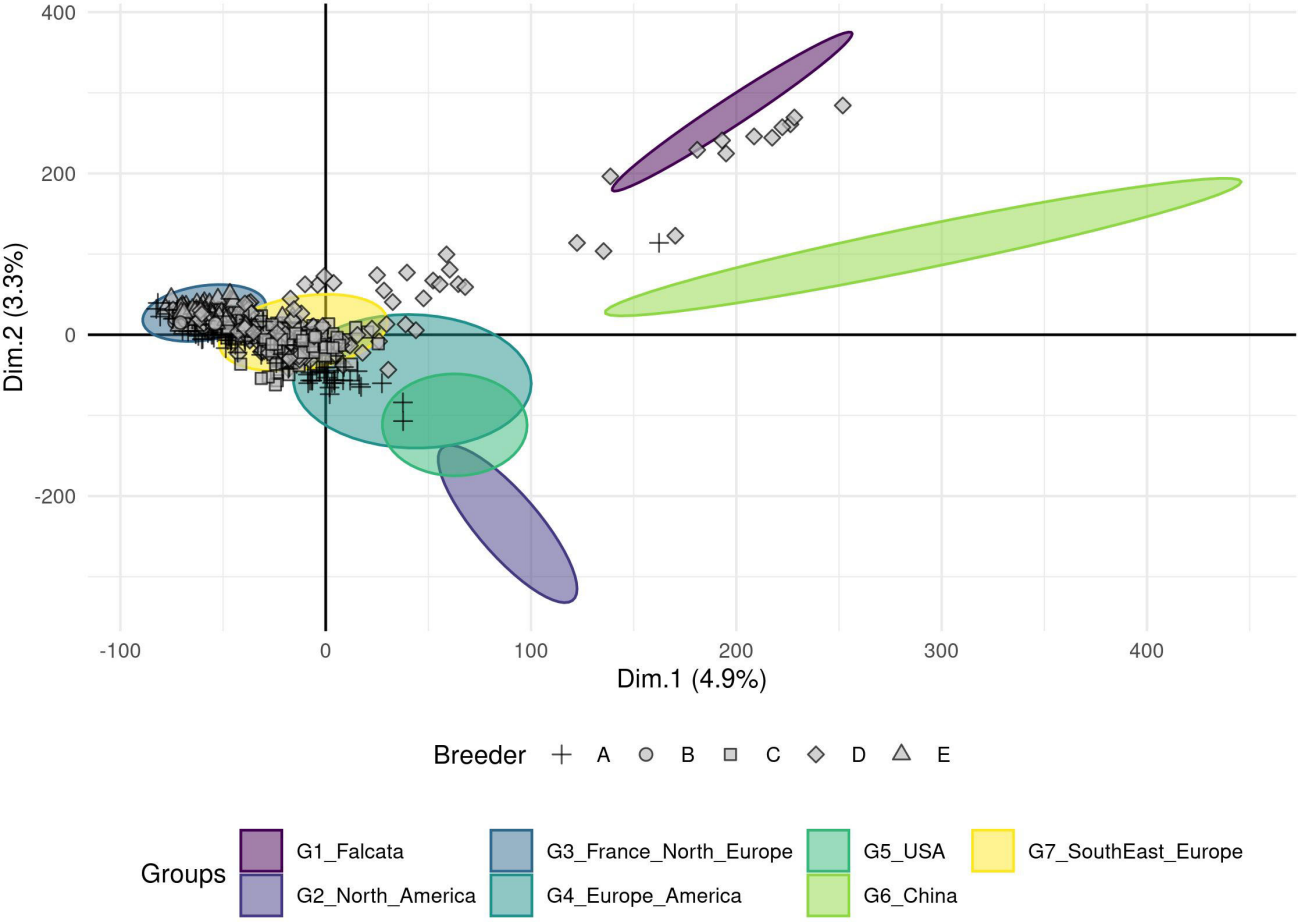
Projection of the 617 European breeders’ accessions on the seven groups (ellipses) obtained from a DAPC analysis using a genotyping dataset of 89 216 SNPs without missing data. A particular point shape represents each breeder.

The breeding material provided by five European breeders was compared to the groups obtained with the worldwide cultivated material (**Figures 3**, **S2** for a detailed view by breeder). Three breeders provided materials with a narrow genetic diversity that were assigned to a single group: accessions of breeders B and E were assigned to group 3 (France and Northern Europe), those of breeder C were assigned to group 7 (Southern and Eastern Europe). The other two breeders provided more diversified genetic materials. The accessions of breeder A covered at least three groups: 3, 7, and 4 (Europe, North and South America). The accessions of breeder D covered groups 3 and 7, and more surprisingly, it seems that some of this material was crossed with *falcata* or possibly Chinese accessions. Nonetheless, the genetic material provided by the five European breeders in this study did not belong to the North American nor the Chinese groups.

**Figure 4:**
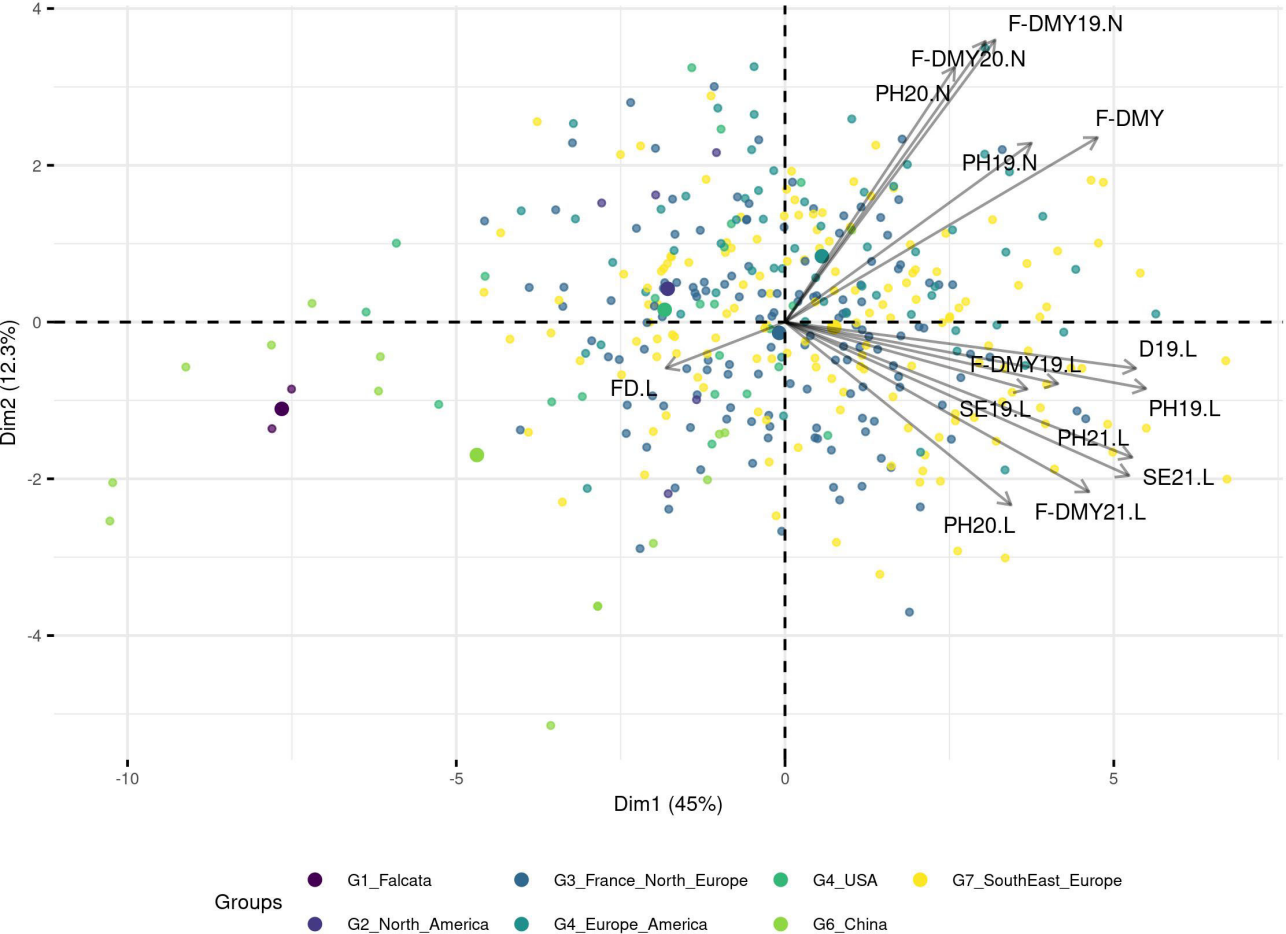
Principal Component Analysis (PCA) based on the phenotypic values, the accessions are colored per DAPC group. The traits are related to Flowering date (FD) and autumn dormancy depending on different measurements in autumn: Dormancy (D), Dry Matter Yield (F-DMY), plant height (PH), Speed of elongation (SE) for two years: 2019 (X19.X) and 2020 (X20.X) in two locations: Lusignan (.L) in France and Novi Sad (.N) in Serbia. F-DMY without letter or number is the Dry Matter Yield adjusted for year and location effects. The biggest points represent the centroids of each cluster.

**Figure 5:**
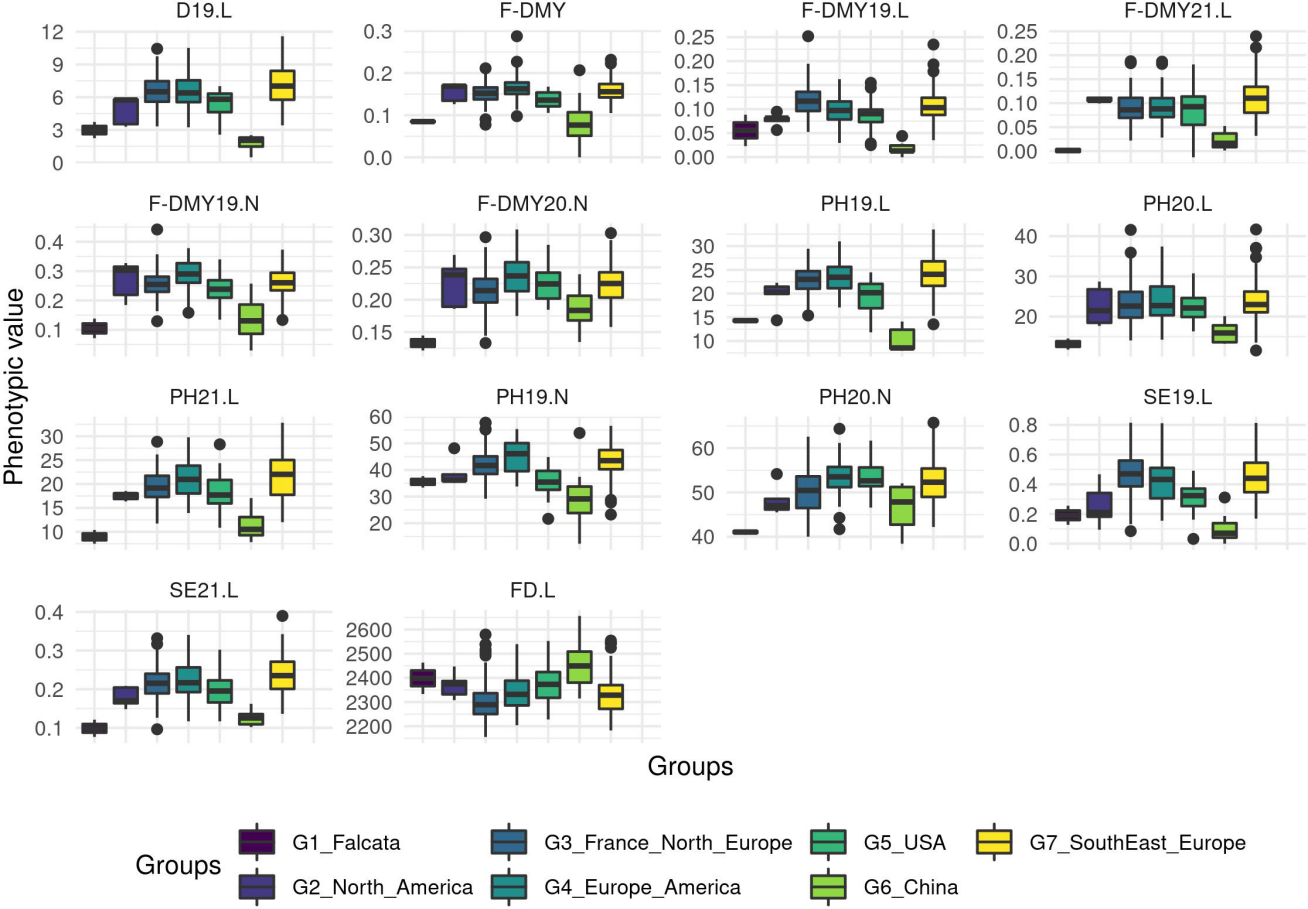
Boxplot of the phenotypic values per group for all the traits related to Flowering date (FD) and autumn dormancy scored from different measurements in autumn. Dormancy (D), Dry Matter Yield (F-DMY), plant height (PH), Speed of elongation (SE) for two years: 2019 (X19.X) and 2020 (X20.X) in two locations: Lusignan (.L) in France and Novi Sad (.N) in Serbia. F-DMY without letter or number is the Dry Matter Yield measured in autumn adjusted for year and location effects.

A PCA based on phenotypic data did not reveal any structure and the genetic groups overlapped with each other (**Figure 4**; for a PCA based on molecular data and coloured per trait, see **Figure S3**). However, when the trait variation was displayed as boxplots per group (**Figure 5**), the group with the Chinese accessions showed the lowest values of the traits linked to fall dormancy (highly dormant) and the highest values for the flowering date. The other groups were relatively similar, except group 1, which contained only two *falcata*-type accessions.

### 3.3 Heritability and genetic correlation

Model estimated variances, heritability and accession mean estimated after phenotypic adjustment, are presented in **Table 3**. Our study showed a wide variation in heritability, ranging from 0.01 for F-DMY20.N to 0.79 for PH20.L that evaluates fall dormancy. F-DMY20.N, PH20.N, PH21.L, and F-DMY21.L showed the lowest heritability (<0.04). These low heritabilities can be explained by a high micro-environmental variance (VarE) and not by the absence of genetic variability (VarG) for the trait. On average, the heritabilities were higher in Lusignan (0.34) than in Novi Sad (0.13). Similarly, the average heritabilities of the traits measured in 2019 (0.48) were higher than those of the traits measured in 2020 (0.09) and 2021 (0.06). Finally, the measurements of SE and PH had higher average heritabilities (0.27) than the measurements of F-DMY (0.18).

**Table 3:** Results of models fit by trait with mean by phenotype, genetic (VarG), spatial by location and year (VarEX.X) and residual (VarR) variances used for heritability estimation (h²).

Traits	mean	VarG	VarE18.L	VarE19.L	VarE20.L	VarE21.L	VarE19.N	VarE20.N	VarR	h²
D19.L	6.62	2.129		0.281					0.5929	0.71
FD.L	2327.82	3285	4481						3809	0.28
F-DMY	0.15	0.00035		0.0000214	0.0000089	0.0036	0.00213	0.00056	0.00181	0.15
F-DMY19.L	0.10	0.0006		0.000105					0.00046	0.52
F-DMY19.N	0.26	0.00186		0.00495					0.00153	0.22
F-DMY20.N	0.22	0.00027			0.02653				0.00167	0.01
F-DMY21.L	0.10	0.00064				0.06298			0.0009	0.01
PH19.L	22.98	9.454		0.5518					1.928	0.79
PH19.N	42.16	22.95		41.2					20.59	0.27
PH20.L	23.27	14.78			32.8				14.37	0.24
PH20.N	51.66	4.441			87.93				52.77	0.03
PH21.L	20.31	11.38				1223			5.707	0.01
SE19.L	0.43	0.00816		0.00159					0.0133	0.35
SE21.L	0.22	0.00136				0.00539			0.00105	0.17

The traits are: flowering date (FD) and fall dormancy (Dormancy: D, plant height: PH, stem elongation rate: SE, Fall Dry Matter Yield: F-DMY) for different locations (Lusignan: L, and Novi Sad: N) over three years of trials (2019: X19.X, 2020: X20.X and 2021: X21.X) or overall (F-DMY).

**Table 4** shows the phenotypic and genetic correlation between traits. All the traits related to fall dormancy presented positive phenotypic and genetic correlations between each other. As expected, FD.L showed negative phenotypic and genetic correlation with all the traits related to fall dormancy. Among the traits related to fall dormancy, the traits measured in Lusignan presented stronger average genetic correlation (0.83) than at Novi Sad (0.78) and stronger than between locations (0.48). Interestingly, PH19.L and PH19.N that were the same trait measured in the same year but in two different locations, presented a genetic correlation of 0.997. These results suggest a low genotype by environment interaction for this trait linked to fall dormancy. Among the fall dormancy traits, F-DMY19.N showed the lowest but still positive genetic correlation (lower than 0.6) with all the other traits.

**Table 4:** Correlation between traits measuring flowering date (FD) and fall dormancy (Dormancy: D, plant height: PH, stem elongation rate: SE, Fall Dry Matter Yield: F-DMY) for different locations (Lusignan: L, and Novi Sad: N) over three years of trials (2019: X19.X, 2020: X20.X and 2021: X21.X) or overall (F-DMY).

	D19.L	F-DMY	F-DMY19.L	F-DMY21.L	PH19.L	PH20.L	PH21.L	SE19.L	SE21.L	F-DMY19.N	F-DMY20.N	PH19.N	PH20.N	FD.L
D19.L		0.738	0.743	0.883	0.930	0.998	0.856	0.848	0.966	0.352	0.599	0.768	0.658	-0.313
F-DMY	0.639		0.768	0.710	0.843	0.806	0.746	0.804	0.776	0.886	0.816	0.919	0.723	-0.239
F-DMY19.L	0.631	0.603		0.578	0.726	0.605	0.553	0.926	0.649	0.554	0.484	0.659	0.319	-0.396
F-DMY21.L	0.553	0.561	0.409		0.828	0.999	0.934	0.700	0.964	0.365	0.553	0.647	0.735	-0.054
PH19.L	0.865	0.666	0.639	0.602		0.995	0.850	0.882	0.897	0.367	0.333	0.997	0.677	-0.292
PH20.L	0.400	0.358	0.309	0.535	0.469		0.746	0.590	0.998	0.107	0.119	0.990	0.844	0.030
PH21.L	0.674	0.600	0.441	0.796	0.707	0.538		0.735	0.993	0.291	0.314	0.703	0.852	-0.104
SE19.L	0.568	0.388	0.455	0.338	0.602	0.178	0.417		0.800	0.215	0.163	0.294	0.150	-0.492
SE21.L	0.698	0.584	0.462	0.748	0.707	0.542	0.926	0.456		0.257	0.297	0.383	0.248	-0.130
F-DMY19.N	0.364	0.707	0.266	0.188	0.429	0.488	0.398	0.538	0.398		0.796	0.706	0.681	-0.403
F-DMY20.N	0.351	0.495	0.202	0.234	0.642	0.730	0.683	0.534	0.654	0.352		0.726	0.995	-0.048
PH19.N	0.484	0.546	0.324	0.304	0.518	0.223	0.417	0.833	0.714	0.528	0.321		0.788	-0.373
PH20.N	0.308	0.347	0.094	0.195	0.276	0.138	0.289	0.505	0.762	0.251	0.636	0.284		-0.038
FD.L	-0.235	-0.248	-0.282	-0.123	-0.227	-0.083	-0.161	-0.221	-0.156	-0.219	-0.066	-0.172	-0.046	

The lower part in grey represents the phenotypic correlation between traits after phenotypic adjustment and the upper part represents the genetic correlation between traits based on the covariance matrix estimated with a multi-trait model.

We found QTL for four out of the eleven traits of this study (**Figure S4**). For D19.L, a single QTL was found on chromosome 8 (chr 8) explaining 14.6% (**Table 5**) of the phenotypic variation. For F-DMY20.N, five QTL were detected: one QTL each on chr 2, 5, 6 and two on chr 7, explaining between 6% and 9% each; overall, they explained 32.1% of the phenotypic variation. For PH19.N, we found six QTL, one on chr 2 and chr 4, two on chr 3 and chr 7, each explaining between 7% and 11.9% of the variation; overall they explained 42.6%. Finally, for F-DMY, we found five QTL on chr 2, and four on chr 3, each explaining between 9.5% and 15.3% of the variation; overall they explained 43.2% of the phenotypic variation. QTL located on the same chromosome were spaced at least 9 Mb apart. No QTL was detected for flowering date. To understand the lack of common QTL between genetically correlated traits, we looked in detail at all the SNPs that were detected as potential QTL by the MLMM method. This iterative method added the potential QTL one by one as a co-factor in the model before estimating which model is the best and thus which are the “true” QTL. The additional data table (**Table S2**) tracked all SNPs selected by each iteration and for each trait. We observed a few cases where QTL for the different traits were located in close proximity. In four cases, the significantly associated SNPs are less than 1000 bp apart. Among these cases, for a pair of SNPs (chr2_12854184 - chr2_12854196), neither of them passed the threshold but were selected by the mlmm method at certain steps, these two SNPs would have an effect on the following traits: PH19.L and F-DMY20.N. In the remaining three cases, one of the two SNPs of the pair was retained as a QTL: chr3_61230828 - chr3_61230888 for PH20.N and F-DMY, respectively; chr3_89061410 - chr3_89061526 for F-DMY and PH19.L, respectively; chr8_51582915 - chr8_51582964 for PH20.N and D19.L. Three SNPs were selected for two different traits: chr2_14385543 for D19.L and F-DMY19.L; chr7_46045300 for PH21.L and SE21.L; chr7_90860527 for PH21.L and SE21.L, but not detected as QTL. **Table S4** shows the distance between the QTL (highlighted) and other non-conserved SNPs that are less than 1000 bp apart. Between the detected QTL, the distance was large and the linkage disequilibrium values were very low, 0.006 on average. However, in cases where the SNPs are in close vicinity, the linkage disequilibrium locally increased strongly. These regions certainly contained QTL related to fall dormancy, but we cannot consider them as such due to our conservative threshold, which allowed us to limit both the number of false positives and a too small number of accessions leading to a low power of detection.

**Table 5:** QTL for autumn dormancy from a GWAS analysis with the MLMM method.

Trait	SNP	r2	r2 Global	Chromosome	Position	Annotation of Chen et al., 2020		Mercedes				Medicago tuncatula			
						Gene	Function	Gene 1	Function	Gene 2	Function	Gene 1	Function	Gene 2	Function
D19.L	chr8_51582964	0.146		chr8.2	51582964	MS.gene035451	ACP-like superfamily; Protein kinase-like domain superfamily	MsNRG001015g01756361	hypothetical protein	MsNRG000350g01027551	hypothetical protein	MtrunA17Chr7g0240251	hypothetical protein		
F-DMY20.N	chr7_53172294	0.090	0.321	chr7.2	53172294	MS.gene010897	Heat shock protein 70kD, peptide-binding domain superfamily	MsNRG000432g01160181	hypothetical protein						
F-DMY20.N	chr7_62988589	0.059		chr7.2	62988589	MS.gene67894	Alcohol dehydrogenase, N-terminal	MsNRG000037g00224451	Putative 2-methylene-furan-3-one reductase			MtrunA17Chr7g0228011	Putative 2-methylene-furan-3-one reductase		
F-DMY20.N	chr6_40691177	0.086		chr6.2	40691177										
F-DMY20.N	chr5_72247110	0.060		chr5.2	72247110	MS.gene98525	Lipid-binding serum glycoprotein, N-terminal ; Lipid-binding serum glycoprotein, C-terminal								
F-DMY20.N	chr2_12854196	0.086		chr2.2	12854196	MS.gene073096	Nucleotide-binding alpha-beta plait domain superfamily ; U2 auxiliary factor small subunit	MsNRG001447g01984221	Putative transcription factor C3H family	MsNRG001933g02106001	Putative transcription factor C3H family	MtrunA17Chr2g0320811	hypothetical protein		
PH19.N	chr3_90724627	0.119	0.426	chr3.2	90724627	MS.gene014383	Tetratricopeptide-like helical domain superfamily	MsNRG000840g01620971	Putative tetratricopeptide-like helical domain superfamily	MtrunA17Chr3g0143411	Putative tetratricopeptide-like helical domain superfamily	MtrunA17Chr3g0143411	Putative tetratricopeptide-like helical domain superfamily		
PH19.N	chr7_90834458	0.106		chr7.2	90834458										
PH19.N	chr7_10758065	0.105		chr7.2	10758065	MS.gene020657		MsNRG143424g02636541	Putative diacylglycerol kinase (ATP)	MsNRG000355g01035221	hypothetical protein				
PH19.N	chr4_5061600	0.080		chr4.2	5064100										
PH19.N	chr3_73810222	0.074		chr3.2	78458544	MS.gene06565	ABC transporter type 1, transmembrane domain	MsNRG000105g00468091	Putative Type I protein exporter	MsNRG000921g01687971	Putative ABC-type xenobiotic transporter	MtrunA17Chr3g0128361	Putative peptidase C78, ubiquitin modifier-specific peptidase 1/ 2	MtrunA17Chr3g0128381	Putative Type 1 protein exporter
PH19.N	chr2_13623107	0.103		chr2.2	13623107	MS.gene69859		MsNRG066413g02467211	Putative protein-serine/threonine kinase CMGC-GSK family						
F-DMY	chr3_44341487	0.130	0.432	chr3.2	44341487	MS.gene027073	WD40/YVTN repeat-like-containing domain superfamily	MsNRG000206g00737911	hypothetical protein	MsNRG000206g00737801	Putative transcription factor WD40-like family				
F-DMY	chr3_89061410	0.095		chr3.2	89061410	MS.gene40203	ACP-like superfamily	MsNRG000661g01450031	Putative thimet oligopeptidase	MsNRG000350g01027551	Putative thimet oligopeptidase	MtrunA17Chr7g0240251	hypothetical protein		
F-DMY	chr3_61230888	0.101		chr3.2	61230888	MS.gene048728	Leucine-rich repeat domain superfamily;F-box domain	MsNRG000832g01614171	Putative F-box domain, leucine-rich repeat domain superfamily, F-box-like domain superfamily	MsNRG000832g01614181	Putative leucine-rich repeat domain superfamily				
F-DMY	chr2_2373521	0.137		chr2.2	2373521	MS.gene36359	Malic enzyme, N-terminal domain superfamily; Malic oxidoreductase	MsNRG000410g01125591	Putative malate dehydrogenase (oxaloacetate-decarboxylating) (NADP(+))	MsNRG000006g00057221	Putative malate dehydrogenase (oxaloacetate-decarboxylating) (NADP(+))	MtrunA17Chr2g0331051	Putative malate dehydrogenase (oxaloacetate-decarboxylating) (NADP(+))	MtrunA17Chr2g0331061	hypothetical protein
F-DMY	chr3_78458544	0.153		chr3.2	78458544	MS.gene06565	ABC transporter type 1, transmembrane domain	MsNRG000105g00468091	Putative Type I protein exporter	MsNRG000921g01687971	Putative ABC-type xenobiotic transporter	MtrunA17Chr3g0128361	Putative peptidase C78, ubiquitin modifier-specific peptidase 1/ 2	MtrunA17Chr3g0128381	Putative Type 1 protein exporter

This table summarizes the exact position of the QTL on the reference genome for each phenotypic trait, the percentage of phenotypic variance explained by the QTL (r^2^) and the available annotation on the cv. XinJiangDaYe reference genome (Chen et al., 2020), the cv. Mercedes genome sequence (Carrère et al., 2020) and the corresponding region on the model species Medicago truncatula (version 5.1.8; Pecrix et al., 2018).

For 14 out of the 17 QTL, genes were found in the 5 kb flanking genomic region. Details are given in **Table 5** and the corresponding annotations are listed in **Table S3**. Some genes encode similar functions, or may be involved in a common biological process. These functional annotations include: drought stress response (2-methylene-furan-3-one reductase (Singh et al., 2022), transcription factor C3H family (Kumar et al., 2019)), growth and development (ABC-type xenobiotic transporter (Verrier et al., 2008), F-box domain (Gupta et al., 2015), malate dehydrogenase (oxaloacetate-decarboxylating) (NADP(+)) (Kujur et al., 2016)) and diverse biological processes (ACP-like superfamily (Zhao et al., 2022), transcription factor WD40-like family (Xu and Min, 2011), and leucine-rich repeat domain superfamily (Liu et al., 2022)). The functions of certain genes linked to our QTL (peptidase C78, ubiquitin modifier-specific peptidase 1/2, thimet oligopeptidase, Type I protein exporter) were not well known in plants in general nor legumes in particular.

### 3.4 Genomic prediction

**Figure 6:**
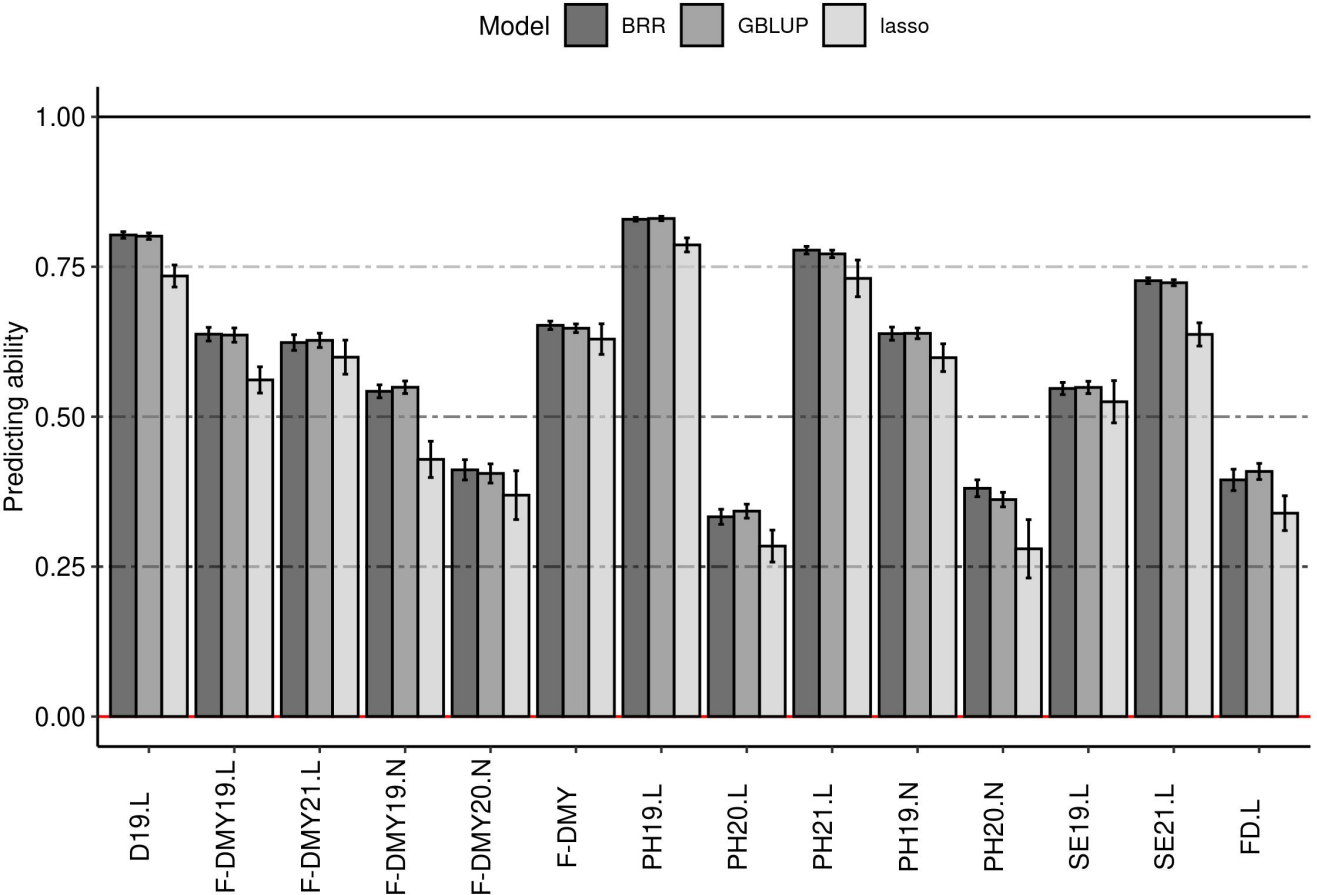
Impact of the model on the predicting ability (y-axis) for phenology traits. Three models were tested: best linear unbiased prediction model (GBLUP), Bayesian Ridge-regression (BRR) and Bayesian Lasso (lasso). The error bars are the standard deviation estimated on 10 repetitions. Traits were: Dormancy (D), Dry Matter Yield (F-DMY), plant height (PH), Speed of elongation (SE) for two years: 2019 (X19.X) and 2020 (X20.X) in two locations: Lusignan (.L) in France and Novi Sad (.N) in Serbia and Flowering date (FD). F-DMY without letter or number is the Dry Matter Yield adjusted for year and location effects.

The predicting ability obtained in this study for flowering date and the traits linked to fall dormancy varied from 0.25 to 0.80, depending on the trait and the method (**Figure 6**). The GBLUP and the BRR method gave equivalent predicting ability (**Figure 6**), the Bayesian Lasso method gave slightly lower prediction quality and a higher standard deviation. The main differences in predicting ability depended on the trait. Fall dormancy in Lusignan showed a higher predicting ability (from 0.50 to 0.80) than in Novi Sad (from 0.25 to 0.68). The results showed less variation between years in Lusignan (2019 and 2021) than in Novi Sad (2019 and 2020). The predicting ability for all traits obtained with GBLUP were above 0.25. The lowest predicting ability on average (above 0.25) were obtained for PH20.L, PH20.N, F-DMY20.N and FD.L. The best predicting abilities (average >0.75) were obtained with D19.L, PH19.L and PH21.L. Other traits had a predicting ability higher than 0.5. Only the results with GBLUP were considered for the rest of the study.

**Figure 7:**
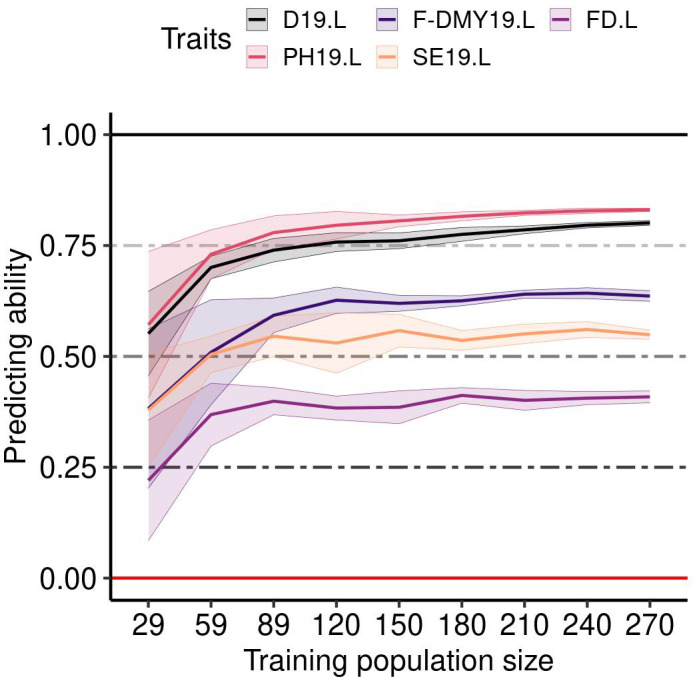
Impact of the training population size on the predicting ability for five traits (Flowering date (FD), Dormancy (D), Dry Matter Yield (F-DMY), plant height (PH), Speed of elongation (SE)) recorded in Lusignan in 2019. On the x-axis, the number of accessions used to train the model to predict a validation population composed of 100 accessions. The accessions were randomly taken among all the clusters. The average predicting ability (ten repetitions) estimated with the spearman correlation between the phenotype and its prediction is the middle solid line, the standard deviation is represented by the two solid lines above and below the middle line and colored by trait.

**Figure 8:**
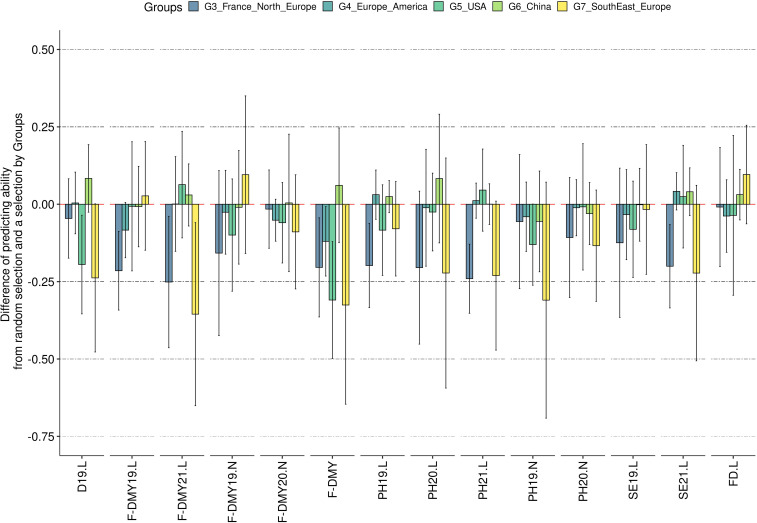
Impact of the training population composition on the predicting ability. The y-axis represents the difference of predicting ability between the predicting ability obtained when no other accessions from the same group were selected in the training population and the predicting ability when some accessions from the same group were in the training population. A negative predicting ability means that the predicting ability obtained with accessions of the same group in the training population was greater than the predicting ability when no accession of the same group was in the training population. The error bars are the standard deviation estimated on 10 repetitions. The traits are represented in the x-axis: Flowering date (FD), Dormancy (D), Dry Matter Yield (F-DMY), plant height (PH), Speed of elongation (SE) for two years: 2019 (X19.X) and 2020 (X20.X) in two locations: Lusignan (.L) in France and Novi Sad (.N) in Serbia. F-DMY without letter or number is the Dry Matter Yield adjusted for year and location effects.

The mean predicting ability (GBLUP method) increased markedly by increasing the size of the training population from 29 to 59 accessions, and reached a plateau with a training population of at least 89 accessions (**Figure 7**). The variance between replicates still decreased in training populations with more than 89 accessions, as shown for five example traits: D19.L, F-DMY19.L, FD.L, PH19.L and SE19.L (**Figure 7**). This observation was consistent across traits and locations (**Figure S5**). Unlike the training population size, which appeared to have a similar effect on all traits, the genetic composition of the training population affected the predicting ability across traits in different ways (**Figure 8**). In order to observe the impact of the training population composition on the predicting ability, **Figure 8** illustrates the difference between a prediction of accessions of one group with a training population including accessions from the other groups and a prediction of the same accessions with a training population of the same size (210) but including accessions from all groups. In the majority of cases, the prediction quality decreased when no accession from the targeted group was included in the training population. Nevertheless, in some cases, the difference was in favour of the prediction across groups for some of the group/traits. The groups 3 (France, Northern Europe) and 7 (Southern and Eastern Europe) seemed to be the most impacted by the absence of their own accessions in the training population. In contrast, group 6 (China) was little impacted by the genetic composition of the training population.

## 4 Discussion

We designed this study firstly to describe the genetic structure among currently grown alfalfa plant materials (‘cultivated material’) and plant breeding materials from the major European breeders (‘breeding material’). Secondly, we wanted to test our hypothesis that breeding programs conducted within a narrow range of fall dormancy could induce a structure within worldwide breeding pools depending on fall dormancy as previously studied by Munjal et al., 2018. The third objective was to study the genetic determinism of major traits related to phenology such as flowering date and fall dormancy, and the possibility to predict it with genomic data.

### 4.1 Genotyping

Our study used a large number of markers (227 092 SNP) with few missing data (<5% per locus), in comparison to other studies in alfalfa (Annicchiarico et al., 2015b; Li et al., 2015b; Nazzicari et al., 2016; Jia et al., 2018; Medina et al., 2020). Previous studies on genomic prediction and genetic diversity showed that a large number of markers allowed to compensate for the amount of missing values (Heslot et al., 2013; Li et al., 2015b). However, a large amount of missing data can lead to a wrong estimation of linkage disequilibrium (Li et al., 2015b) and as a consequence to a biased estimation of the genomic relationship between accessions (Schopp et al., 2017). Genotypic imputation has shown promising results to complete missing values in different species (Marchini et al., 2007; Howie et al., 2009; Marchini and Howie, 2010; Daetwyler et al., 2011; Faville et al., 2018; Pégard et al., 2019). So far, the imputation methods proposed for tetraploid species (Nazzicari et al., 2016; Bastien et al., 2018) have shown interesting results to impute individual genotyping (allele dosage) but not for pool sequencing (allele frequency). These methods are not effective enough to be reliable and routinely used. The development of a method to impute genotypes based on population allele frequencies of tetraploid species could increase the number of useful markers. This would be of particular interest for alfalfa, which showed a very rapid decay of linkage disequilibrium between 1 kb and 20 kb in our case and in several other studies (Herrmann et al., 2010; Li et al., 2014). Indeed, in case of a short LD, a large number of SNPs is required to capture QTL and to obtain a good genomic prediction quality (Wientjes et al., 2013; Liu et al., 2015). A short LD can also influence the prediction methods that use LD to connect to putative QTL by feature selection (e.g., BayesB, Bayes Lasso) and decrease their effectiveness because of inaccurate LD estimation (Habier et al., 2007; Shengqiang et al., 2009; Jannink et al., 2010).

### 4.2 Diversity

With the large number of SNPs that we obtained, a genetic structure was revealed among cultivated material. In previous studies conducted with a limited number of SSR, AFLP or RAPD markers, genetic structure was observed only when a wide genetic diversity was studied (Crochemore et al., 1996; Riday et al., 2003; Qiang et al., 2015) but not when the diversity was restricted to breeding material (Flajoulot et al., 2005; Annicchiarico et al., 2016; Herrmann et al., 2018). We identified seven genetic groups among which two groups were clearly separated from the others. One group consisted of two accessions related to ssp *falcata*. This separation is consistent with another study showing that the ssp *falcata* are clearly separated from cultivated alfalfa (Li et al., 2014). The other group consisted of 15 Chinese accessions. Their clear separation from American and European accessions is more marked in our study than the separation observed in previous studies with SSR markers (Qiang et al., 2015) or genomic markers (Chen et al., 2020; Long et al., 2022). Within accessions of Europe, North and South America, a clear geographic structure was also obtained with partial overlap of the groups, finally showing a continuum. As all American germplasm originates from the “Ancient World”, the complete overlap of European and American genetic diversity was expected, as also found by (Shen et al., 2020). Two reasons could explain our results: (1) some diversity from the “Ancient World” was not represented in our study, such as that of North Africa and Near or Middle East, (2) the selective pressure exerted by American breeders or a genetic drift generated a shift in the diversity. The wide range of dormancy within American materials is not in favour of the first explanation.

When using phenotypic traits related to fall dormancy and flowering date, no structure was observed. Except some of the Chinese accessions that showed a high fall dormancy, the other groups showed similar range of fall dormancy. Fall dormancy was shown to be a good indicator for genetic structure among accessions when the widest range of diversity is studied (Li et al., 2014) but was less efficient within intermediate range of fall dormancy, as studied here. Our results seemed to refute our hypothesis that preferential crossing within dormancy groups and strong selection pressure on dormancy could have induced genetic structure.

The majority of ‘breeding material’ provided by European breeders was genetically close to the groups that contained European ‘cultivated material’. This indicates that cultivated material from North and South America and China are either not introduced in European breeding programs, or, if introduced, not retained after selection steps. This observation is an invitation to go more deeply into a phenotypic analysis of the cultivated material to exploit them in European, American and Chinese breeding programs.

### 4.3 GWAS

We detected several QTL for traits related to fall dormancy on chromosomes 2, 3, 6, 7 and 8. Some of them explained a high percentage of phenotypic variation (>14%). Previous studies already evidenced QTL for fall dormancy. In a first attempt, QTL were found in a mapping population (Brouwer et al., 2000) but the assignment of linkage groups to current physical maps is not available. More recently, also in mapping populations, QTL were found for fall dormancy traits recorded in several environments on chr 1 and 7 (Li et al., 2015a), chr 1, 2, 3, 4, 5, 6, 7 (Adhikari et al., 2018), and chr 1, 7, 8 (Pecetti et al., 2021). In a GWAS, a QTL was found in a region of chr 7 that contained a Flowering locus T gene (MsFTa2), known to be part of the flowering pathway (Shen et al., 2020) and four QTL were found on chr 2, 3, 5, 6 (Long et al., 2022). A precise comparison of QTL positions was difficult because different genome references were used by the respective authors.

### 4.4 Genomic prediction

First of all, this study showed that it was possible to reach a high predicting ability (>0.75 for D19.L, PH19.L and PH21.L) for phenology related traits. However, this was not observed for all traits, the prediction capacity was around 0.30 for some traits (F-DMY20.N, PH20.L, PH20.N and FD.L). In our study, the difference between traits depended mainly on the year of measurement, with the year 2020 having a lower predicting ability on average, and on the location of measurement, with the prediction of traits measured in Novi Sad (Serbia) being less accurate than that measured in Lusignan (France). We do not have a clear explanation for these results, we suppose that it may be related to the difference between the continental climate (Novi Sad) and the oceanic climate (Lusignan) that is warmer in fall allowing a higher potential growth and therefore a better distinction of the fall dormancy of the accessions. Heritability was often presented (Luan et al., 2009; Lorenz et al., 2011; Clark et al., 2012; Kaler et al., 2022) as a factor that explained the difference for predicting ability between traits. In the present study, the quality of prediction could differ greatly for similar estimated heritability. This difference might be explained by the way heritability was expressed in our study, the variance explained by the spatial effect was integrated in the heritability formula, to avoid inflated heritabilities (Heckerman et al., 2016). Indeed, in **Supplementary Figure S6**, we have compared the relationship between predicting ability and heritability with and without taking into account the spatial variance in the heritability estimation. This showed that without taking into account the spatial variance in the estimation of heritability, the relationship between predicting ability and heritability was the same as that expressed in several studies (Luan et al., 2009; Lorenz et al., 2011; Clark et al., 2012; Kaler et al., 2022). However, (Heckerman et al., 2016) emphasized the importance of modelling environmental effects in the estimation of heritability and that the inflation was not stable for all traits, what was observed in our study as well. Even if the difference in heritability between traits did not allow to explain the difference in prediction quality, the traits with a high genetic correlation (D19.L-PH19.L- F-DMY19.L-PH21.L-SE21.L and F-DMY20.N-PH20.N) showed a similar predicting ability.

In this study, the different GP models showed similar results, which was expected for GBLUP and BRR, but not necessarily for the Bayesian Lasso, as the latter selected the SNPs having an effect in the model and was supposed to perform better in the case of strong QTL effects (Meher et al., 2022). However, our results were consistent with those obtained in other studies in alfalfa for various trait (Annicchiarico et al., 2015b; Biazzi et al., 2017; Jia et al., 2018; Medina et al., 2020; Zhang et al., 2023).

Similar to other authors (Nakaya and Isobe, 2012; Tayeh et al., 2015; Cericola et al., 2017), increasing the number of accessions in the training population increased the prediction quality until stabilisation occurred with training populations of 89 accessions. The additional accessions added after this point were useful in reducing the variation due to sampling. By setting the size of the training and validation populations, we ensured that this did not affect the quality of the prediction between the groups. The same applied to the composition of the training population (Lorenz and Smith, 2015; Tayeh et al., 2015; Norman et al., 2018; Akdemir and Isidro-Sánchez, 2019; Pégard et al., 2020). The predicting ability observed in our study was higher and less variable when the training and validation populations were related and when all the groups were represented in the training population, than in the case of across groups prediction.

Our study has shown that for traits such as fall dormancy or flowering date, we can achieve good genomic prediction quality in a diverse panel. These results, combined with other studies on alfalfa (Annicchiarico et al., 2015b; Biazzi et al., 2017; Jia et al., 2018; Andrade et al., 2022; Zhang et al., 2023), show that genomic selection is an interesting and efficient lever in alfalfa breeding. It is possible to rethink the alfalfa improvement scheme by using genomic prediction. It allows to play with several genetic gain parameters such as selection intensity, cycle length and genetic diversity. The value of genomic prediction has been demonstrated, even with a low predicting ability, to increase genetic gain compared to the current selection method (Annicchiarico et al., 2017). Currently, breeders have several breeding populations, as they tend to select within a fall dormancy group. Our study shows that with genomic prediction, this selection process can be simplified by mixing different fall dormancy groups within a single breeding population. Varieties can be selected for fall dormancy during the breeding cycle using molecular markers. Our results also show that flowering date can be predicted and used to optimise seed crosses between parents to increase genetic gain and manage genetic diversity, as has been proposed in other species (Tiret et al., 2021). Advances in genomics-assisted breeding offer exceptional prospects for improving alfalfa’s performance.

## Data availability statement

The datasets presented in this study can be found in online repositories. The names of the repository/repositories and accession number(s) can be found in the article/**Supplementary Material**.

## Author contributions

MP contributed to the genotyping bioinformatics pipeline, conceived and performed the analyses and wrote the paper draft. SD performed DNA extraction, prepared libraries for sequencing. FS contributed to data collection. DK conceived the project with BJ. DM defined the trial experiments and plant material with BJ and coordinated data collection in Novi Sad. MZ set up the experimental augmented design. TR helped with bioinformatics analysis. PB covered the aspects of GWAS and genomic prediction on the project. BJ conceived the project and obtained financial support, contributed to data collection and analyses. All authors participated to the draft and revision of the manuscript and approved the final version. All authors contributed to the article and approved the submitted version.
