# Supplementary material for: Genome-wide genotyping data renew knowledge on genetic diversity of a worldwide alfalfa collection and give insights on genetic control of phenology traits
Source: Front Plant Sci. 2023 Jul 5;14:1196134. doi: 10.3389/fpls.2023.1196134 (PMC10354441; doi:10.3389/fpls.2023.1196134)
Supplement: Supplementary file 1 [file DataSheet_1.docx]

Supplementary Material

Genome-wide genotyping data renew knowledge on genetic diversity of a worldwide alfalfa collection and give insights on genetic control of phenology traits

Marie Pégard ^1*^, Philippe Barre ^1^, Sabrina Delaunay^1^, Fabien Surault^1^, Djura Karagić^2^, Dragan Milić^2,3^, Miroslav Zorić^2^, Tom Ruttink^4^, and Bernadette Julier^1^

^1^ INRAE P3F, 86600 Lusignan, France

^2^ IFVCNS, Novi Sad, Serbia

^3^ CIMMYT, Carretera México-Veracruz Km. 45, El Batán, Texcoco, México, C.P. 56237

^4^ ILVO Plant Science Unit, 9090 Melle, Belgium

*** Correspondence:**Marie Pégard
marie.pegard@inrae.fr

# Supplementary Figures


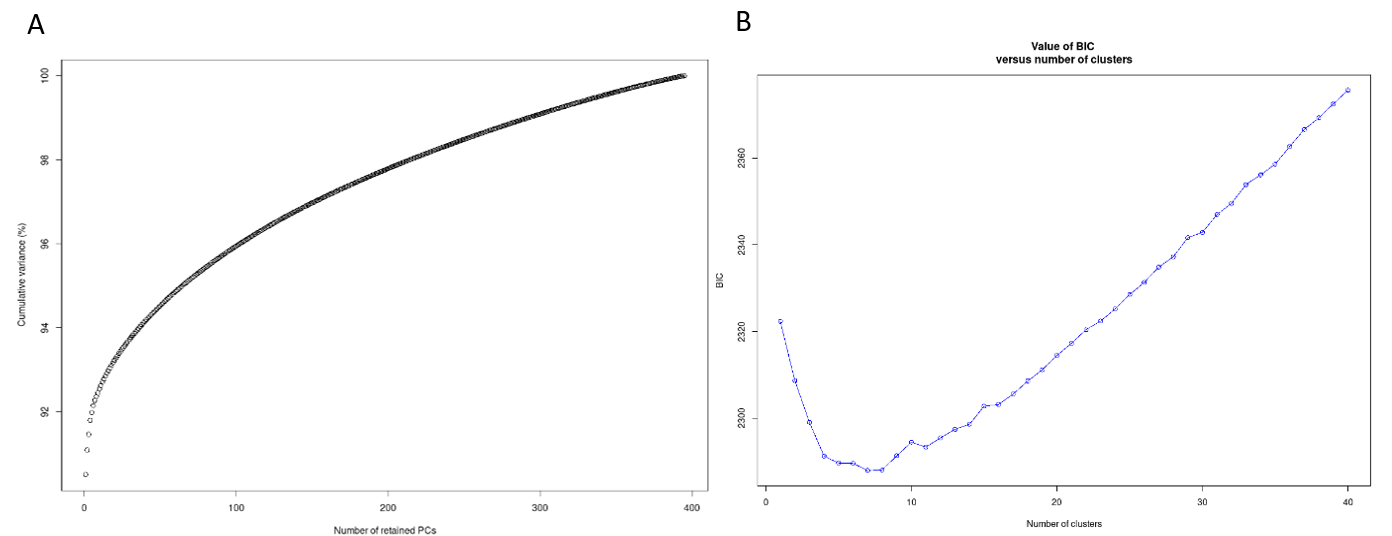


**Supplementary Figure 1.** Results of DAPC clustering. (A) Explained variance (%) depending on the number of Principal Components (PC). (B) BIC value depending on the number of clusters.


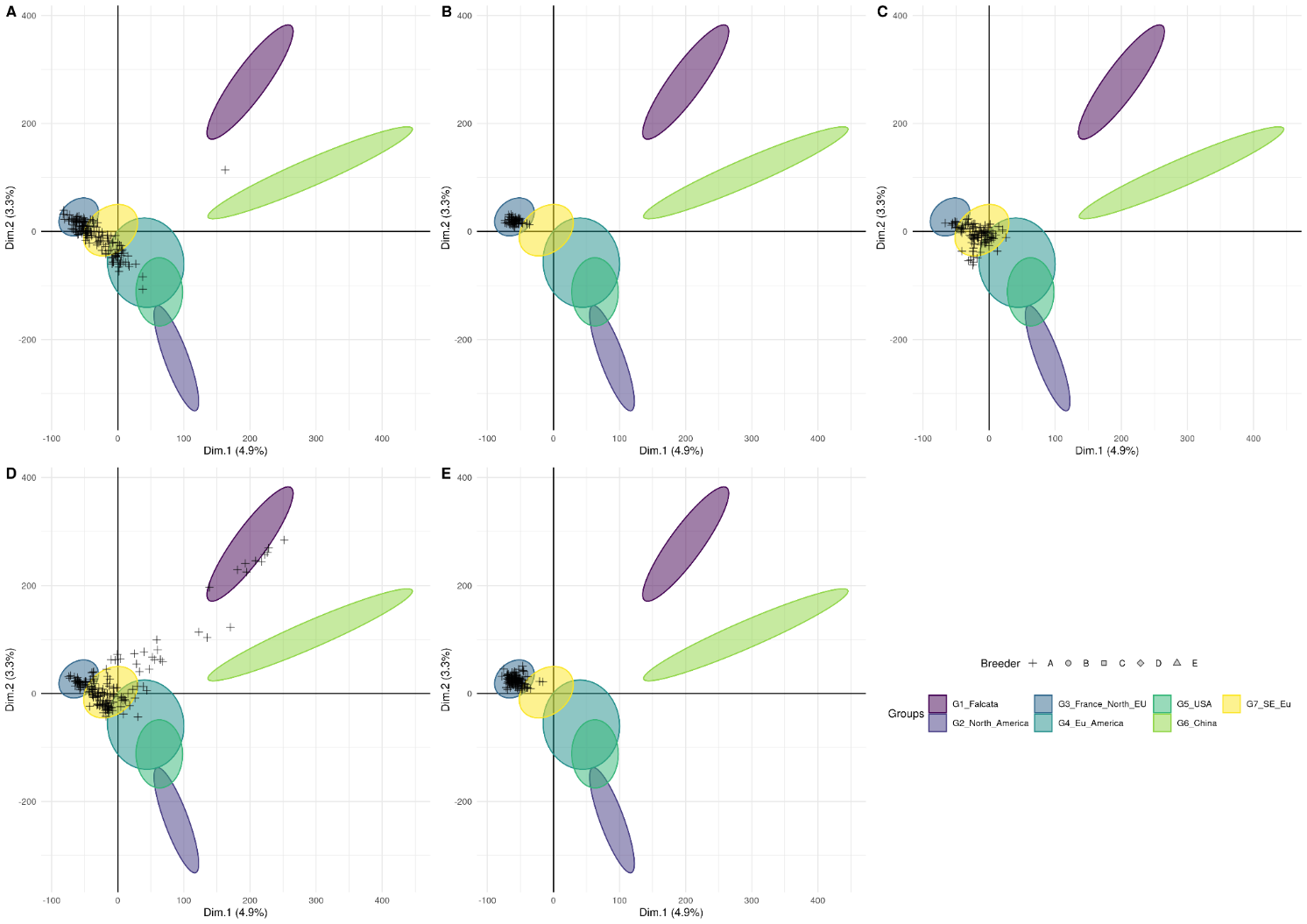


**Supplementary Figure 2.** Projection of the European breeders’ genetic material on the seven groups obtained from a DAPC analysis using a genotyping dataset of 118 421 SNP without missing values. A-E: breeders A to E.

**
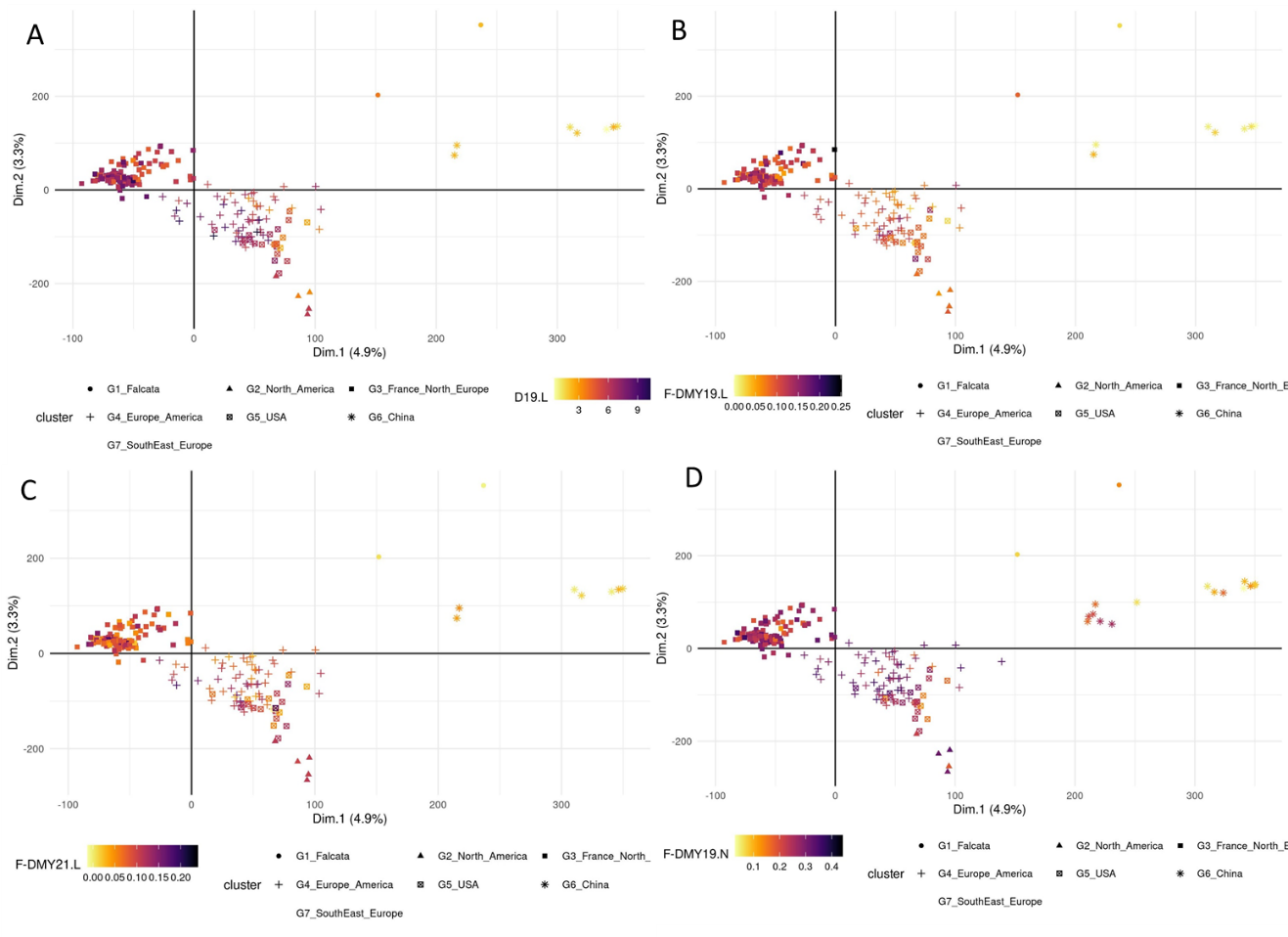

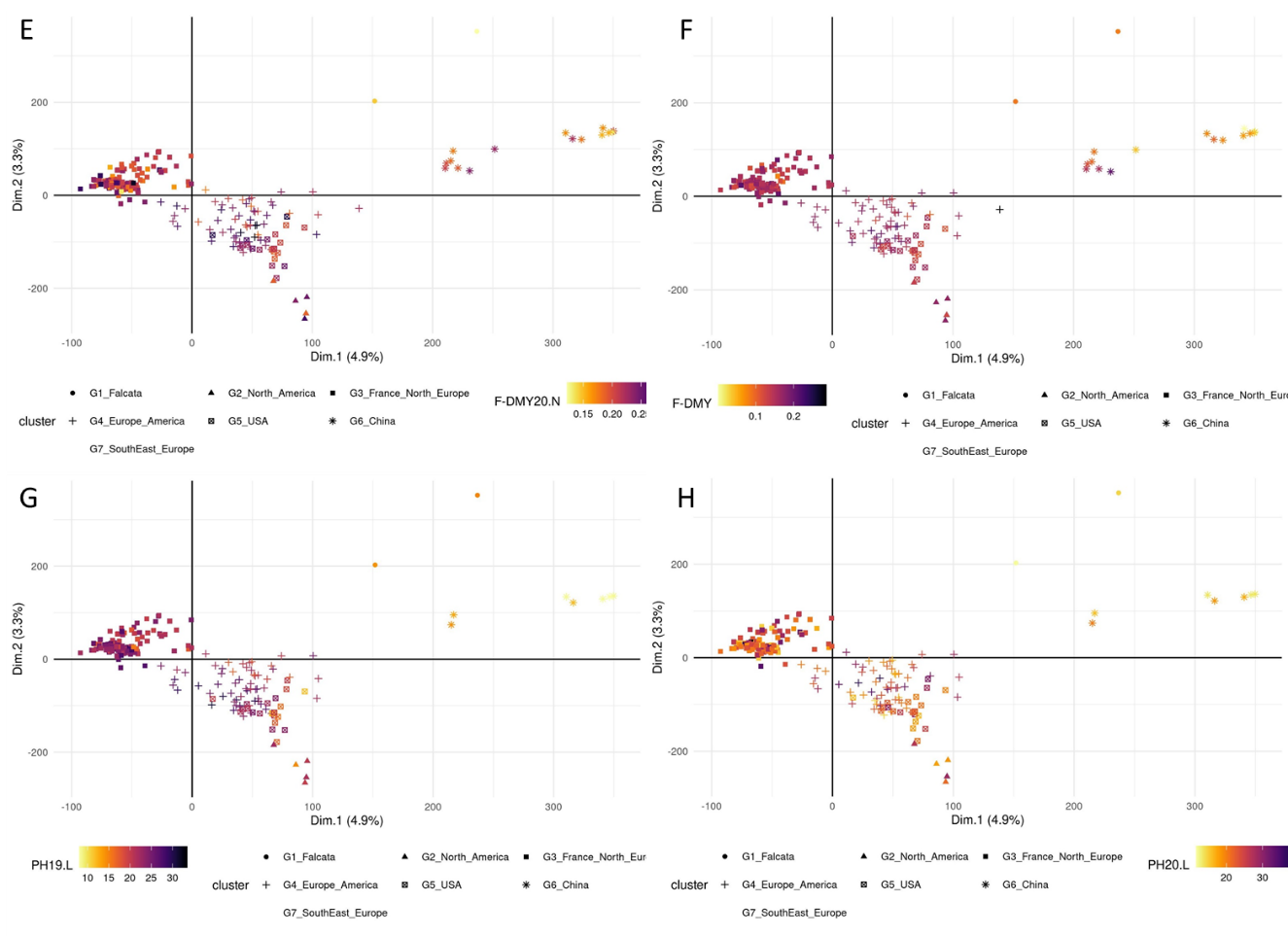
**

**
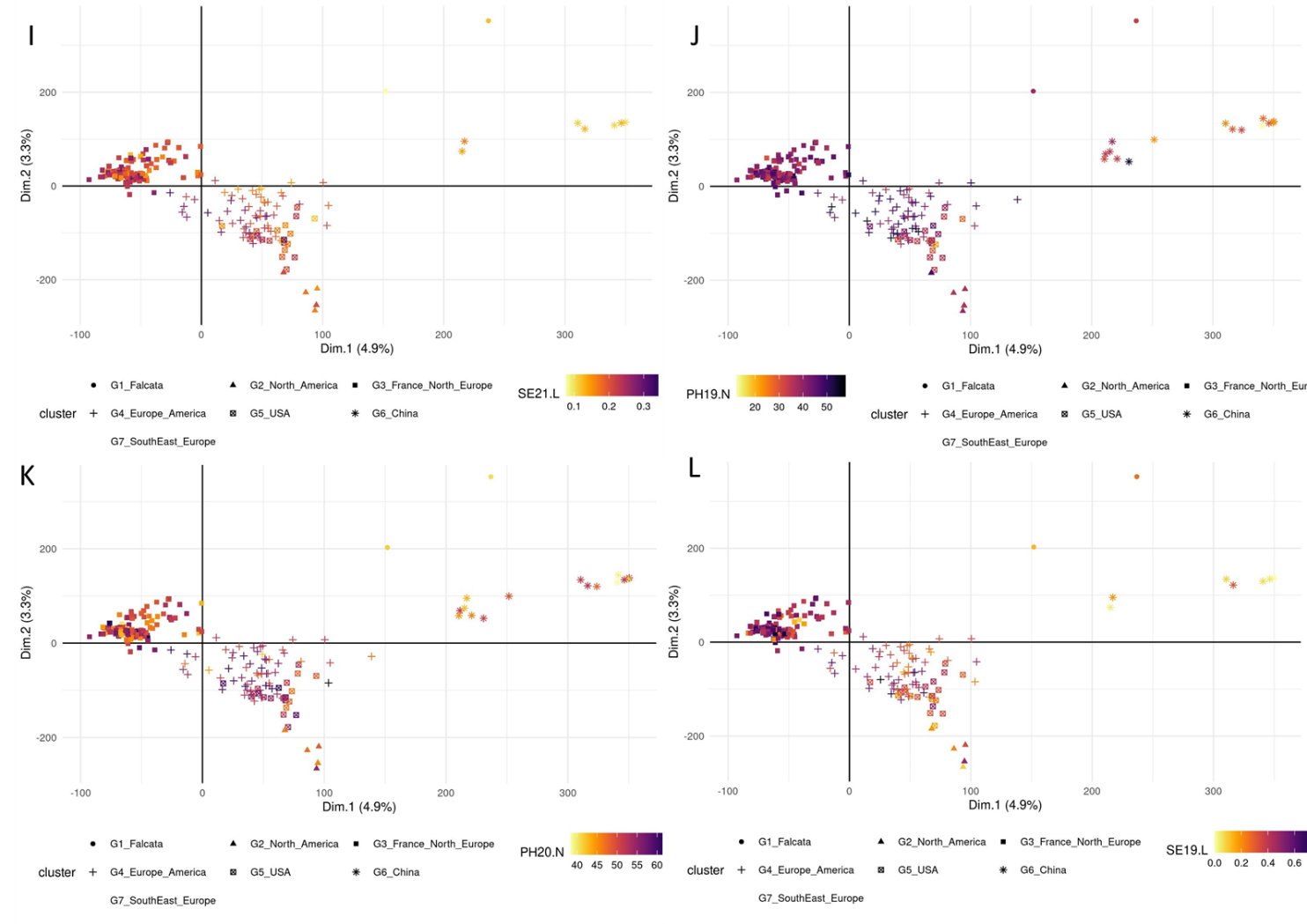
**

**
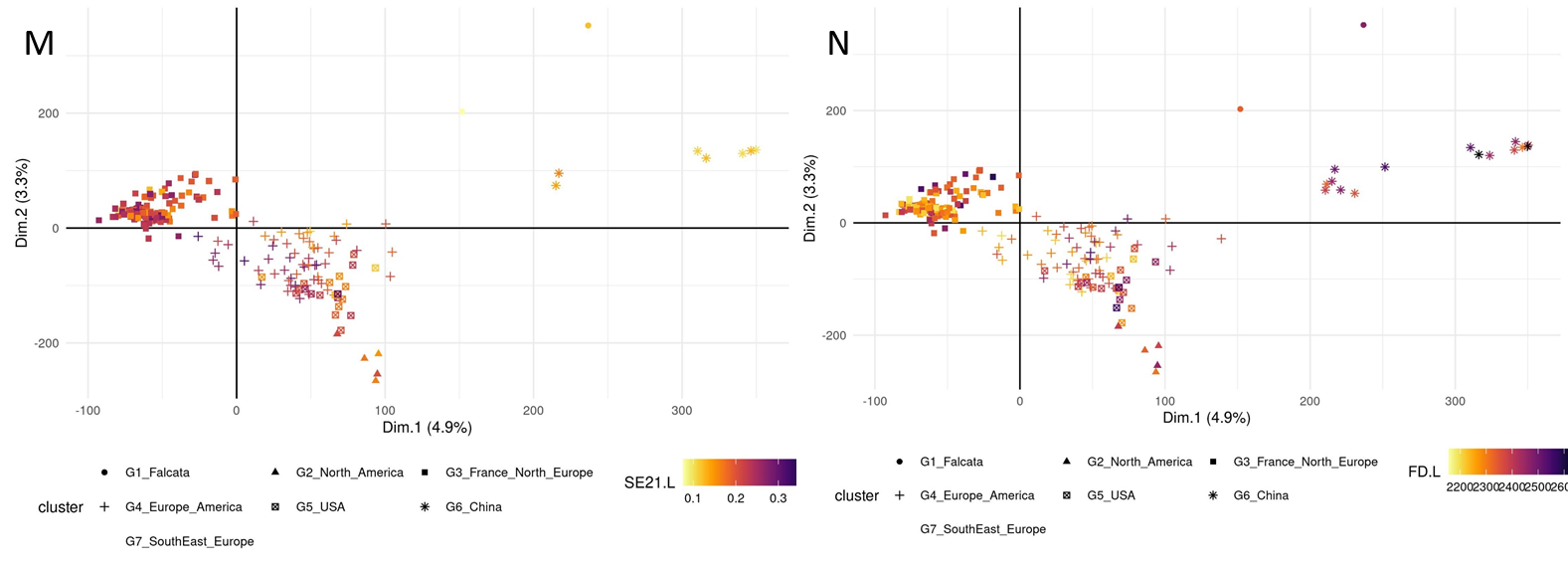
**

**Supplementary Figure 3.** Principal component analysis based on the SNP, the accessions are colored depending on their values for each trait. The traits are related to Flowering date (FD) and autumn dormancy depending on different traits measured in autumn: Dormancy (D), Dry Matter Yield (F-DMY), plant height (PH), Speed elongation (SE) for two years 2019 (X19.X) and 2020 (X20.X) in two locations : Lusignan (.L) in France and Novi Sad (.N) in Serbia. F-DMY without letter or number is the Dry Matter Yield adjusted for year and location effect. The biggest points represents the centroids of each cluster. A : D19.L; B : F-DMY19.L; C : F-DMY21.L; D : F-DMY19.N; E : F-DMY20.N ; F : F-DMY ; G : PH19.L; H : PH20.L; I : PH21.L ; J : PH19.N ; K : PH20.N ; L : SE19.L; M : SE21.L; N : FD.L.


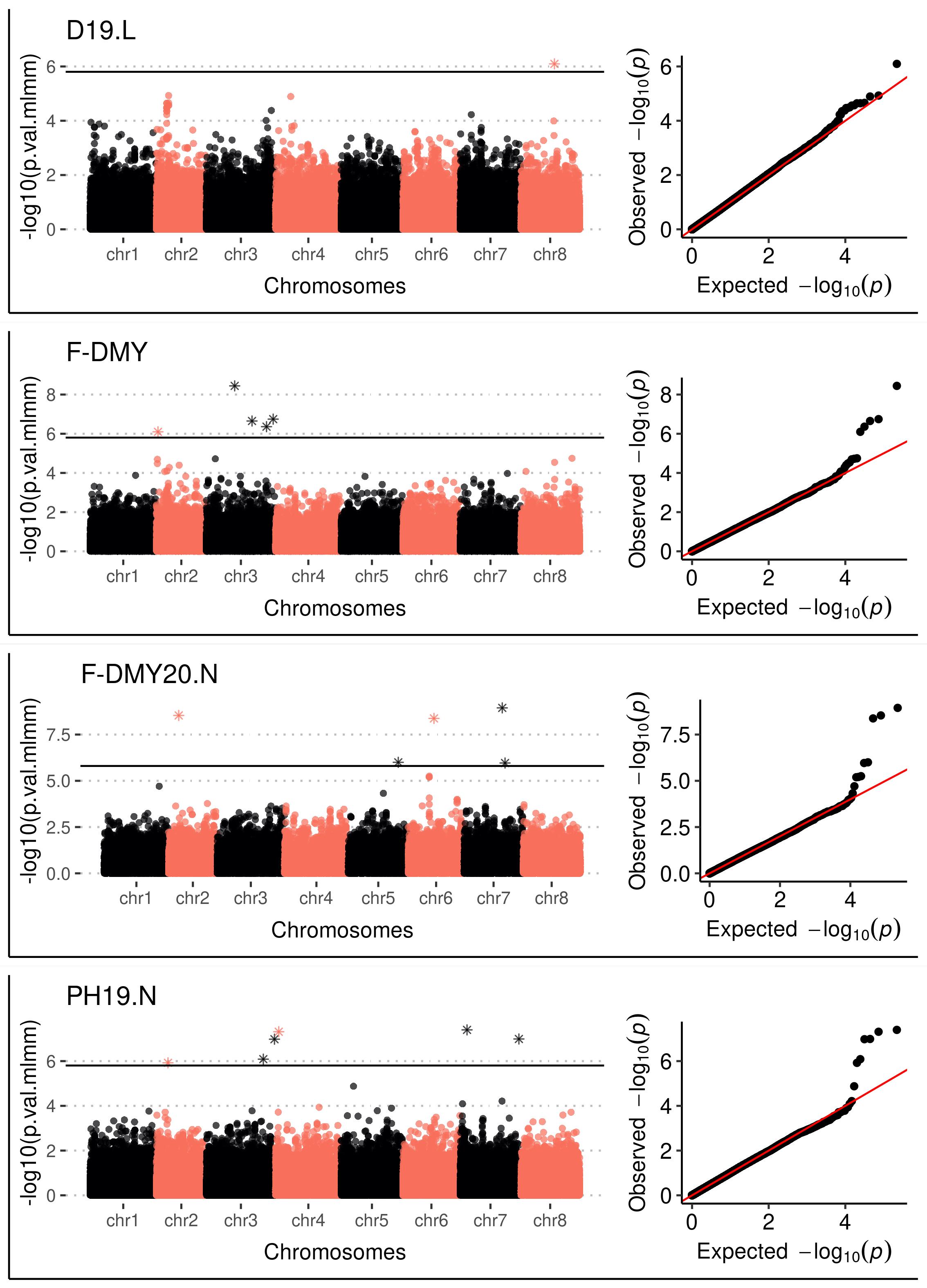


**Supplementary Figure 4.** Manhantan plot and QQplot of the p-value estimated with the MLMM method for three traits. Dry Matter Yield (F-DMY), plant height (PH), Speed elongation (SE)) recorded in Lusignan (.L) and Novi Sad (.N) in autumn 2019, 2020 and 2021.

**
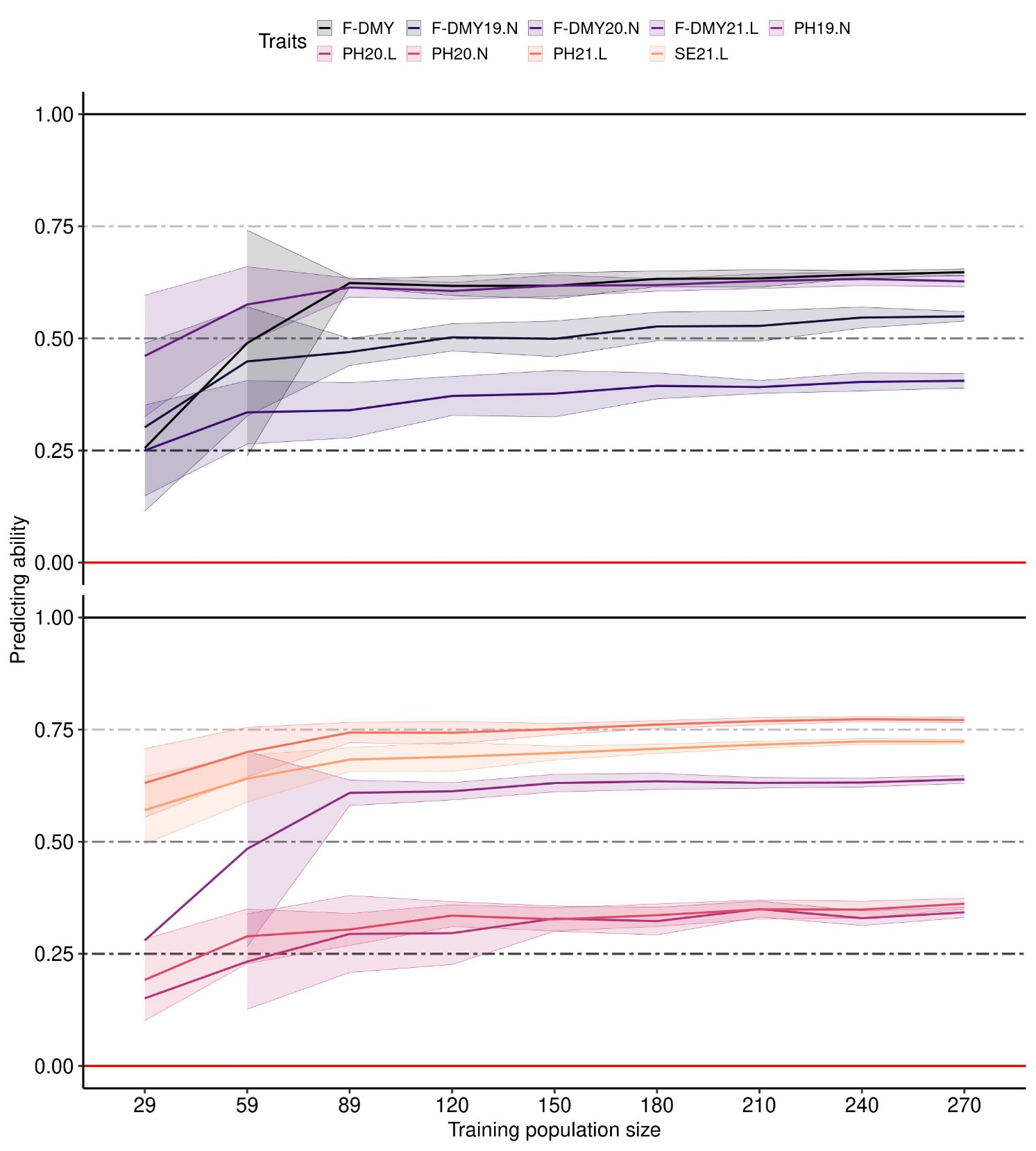
**

**Supplementary Figure 5.** Impact of the training population size on the predicting ability of traits: Dry Matter Yield (F-DMY), plant height (PH), Speed elongation (SE)) recorded in Lusignan (.L) and Novi Sad (.N) in autumn 2019, 2020 and 2021. On the x-axis, the number of accessions used to train the model to predict a validation population composed of 100 accessions. The accessions were randomly taken among all the clusters. The average predicting ability (ten repetitions) estimated with the spearman correlation between the phenotype and its prediction is the middle solid line, the standard deviation is represented by the two solid lines above and under the middle line and colored by trait.

**Supplementary Figure 6.** Relationship between heritability and the average over ten repetitions predicting ability for each trait depending on the method to estimate heritability: (A) with the variance explained by the spatial effect (Heckermann et al., 2016), (B) without the variance explained by the spatial effect. Dry Matter Yield (F-DMY), plant height (PH), Speed elongation (SE) recorded in Lusignan (.L) and Novi Sad (.N) in 2019, 2020 and 2021. The dotted line represent the predicting ability depending on the heritability.

# Supplementary Tables

**Supplementary Table 1.** Description of the trial in the two locations

| **Description** | **Novi Sad** | **Lusignan** |
| --- | --- | --- |
| Length of a single plot (m) | 6 | 4.5 |
| Width of a single plot (m) | 1 | 1.12 |
| Number of plot rows in the trial | 10 | 10 |
| Number of plot columns in the trial | 44 | 44 |
| Number of rows sown in a single plot | 10 | 8 |
| Distance between the rows within a plot (m) | 0.1 | 0.14 |
| Distance between the rows between adjacent plots (m) | 0.4 | 0.4 |
| Sowing depth (m) | 0.015 | 0.01 |
| Sowing density (kg of seeds / ha) | 20 | 20 |

**Supplementary Table 2.** SNPs (snp name) detected as potential QTL at each step of the mlmm method for each traits. The traits are : flowering date (FD) and fall dormancy (Dormance : D,height :PH, stem elongation rate : SE, Fall Dry Matter Yield : F-DMY) for different locations (Lusignan: L and Novi Sad: N) over three years of trials (2019 : X19.X, 2020 : X20.X and 2021 : X21.X) or overall (F-DMY). step is the step were the SNP was selected by the mlmm method, selected step represent the step selected depending on the threshold. The position of the SNP is given by the chromosome (chr) and the position on that chromosome (pos). A distance in base (dist) with the SNP and the next on on the same chromosome was calculated. Depending on that distance, the snp name is colored in yellow if the distance is lower than 1000 bp ; in green when the same SNP is selected for two different traits. The SNPs detected as QTL by the method MLMM and are represent in bold, italic and underlined.

| ***Traits*** | | ***step*** | ***selected step*** | ***snp name*** | ***chr*** | ***pos*** | ***dist*** |
| --- | --- | --- | --- | --- | --- | --- | --- |
|  | D19.L | 6 | 2 | chr1_11570366 | chr1 | 11570366 |  |
|  | D19.L | 9 | 2 | chr1_19446704 | chr1 | 19446704 | 7876338 |
|  | F-DMY19.L | 6 | 1 | chr1_23848836 | chr1 | 23848836 | 4402132 |
|  | PH20.L | 6 | 1 | chr1_31761715 | chr1 | 31761715 | 7912879 |
|  | PH21.L | 9 | 1 | chr1_32978015 | chr1 | 32978015 | 1216300 |
|  | F-DMY19.N | 7 | 1 | chr1_33776940 | chr1 | 33776940 | 798925 |
|  | SE21.L | 3 | 1 | chr1_36798852 | chr1 | 36798852 | 3021912 |
|  | PH19.N | 10 | 7 | chr1_45301674 | chr1 | 45301674 | 8502822 |
|  | FD.L | 9 | 1 | chr1_46148712 | chr1 | 46148712 | 847038 |
|  | PH21.L | 5 | 1 | chr1_53201321 | chr1 | 53201321 | 7052609 |
|  | F-DMY19.N | 8 | 1 | chr1_53852948 | chr1 | 53852948 | 651627 |
|  | PH21.L | 6 | 1 | chr1_58038477 | chr1 | 58038477 | 4185529 |
|  | F-DMY19.N | 10 | 1 | chr1_64385175 | chr1 | 64385175 | 6346698 |
|  | PH20.L | 5 | 1 | chr1_67781881 | chr1 | 67781881 | 3396706 |
|  | F-DMY19.L | 9 | 1 | chr1_83857341 | chr1 | 83857341 | 16075460 |
|  | D19.L | 7 | 2 | chr2_1012142 | chr2 | 1012142 |  |
|  | F-DMY | 8 | 6 | chr2_1375867 | chr2 | 1375867 | 363725 |
|  | ***F-DMY*** | ***6*** | ***6*** | ***chr2_2373521*** | ***chr2*** | ***2373521*** | ***997654*** |
|  | SE19.L | 8 | 1 | chr2_3236671 | chr2 | 3236671 | 863150 |
|  | PH20.L | 7 | 1 | chr2_5505105 | chr2 | 5505105 | 2268434 |
|  | PH19.L | 4 | 1 | chr2_12854184 | chr2 | 12854184 | 7349079 |
|  | F-DMY20.N | 8 | 6 | chr2_12854196 | chr2 | 12854196 | 12 |
|  | ***PH19.N*** | ***7*** | ***7*** | ***chr2_13623107*** | ***chr2*** | ***13623107*** | ***768911*** |
|  | PH21.L | 2 | 1 | chr2_14275519 | chr2 | 14275519 | 652412 |
| D19.L | F-DMY19.L | 3-3 | 2-1 | chr2_14385543 | chr2 | 14385543 | 110024 |
|  | PH20.N | 8 | 1 | chr2_20645909 | chr2 | 20645909 | 6260366 |
|  | F-DMY | 9 | 6 | chr2_29187001 | chr2 | 29187001 | 8541092 |
|  | PH20.L | 4 | 1 | chr2_39106501 | chr2 | 39106501 | 9919500 |
|  | PH19.L | 5 | 1 | chr2_54459033 | chr2 | 54459033 | 15352532 |
|  | F-DMY21.L | 5 | 1 | chr2_62705700 | chr2 | 62705700 | 8246667 |
|  | SE19.L | 10 | 1 | chr2_62777426 | chr2 | 62777426 | 71726 |
|  | PH21.L | 3 | 1 | chr2_70853120 | chr2 | 70853120 | 8075694 |
|  | F-DMY19.N | 5 | 1 | chr3_1400787 | chr3 | 1400787 |  |
|  | F-DMY19.L | 5 | 1 | chr3_7568258 | chr3 | 7568258 | 6167471 |
|  | F-DMY19.N | 6 | 1 | chr3_9301739 | chr3 | 9301739 | 1733481 |
|  | SE19.L | 9 | 1 | chr3_23667516 | chr3 | 23667516 | 14365777 |
|  | F-DMY19.L | 8 | 1 | chr3_28887014 | chr3 | 28887014 | 5219498 |
|  | F-DMY19.N | 2 | 1 | chr3_32484837 | chr3 | 32484837 | 3597823 |
|  | PH20.N | 7 | 1 | chr3_36979545 | chr3 | 36979545 | 4494708 |
|  | ***F-DMY*** | ***3*** | ***6*** | ***chr3_44341487*** | ***chr3*** | ***44341487*** | ***7361942*** |
|  | F-DMY21.L | 6 | 1 | chr3_51632025 | chr3 | 51632025 | 7290538 |
|  | F-DMY19.L | 10 | 1 | chr3_53971209 | chr3 | 53971209 | 2339184 |
|  | SE21.L | 10 | 1 | chr3_60359039 | chr3 | 60359039 | 6387830 |
|  | PH20.N | 6 | 1 | chr3_61230828 | chr3 | 61230828 | 871789 |
|  | ***F-DMY*** | ***5*** | ***6*** | ***chr3_61230888*** | ***chr3*** | ***61230888*** | ***60*** |
|  | SE21.L | 4 | 1 | chr3_64501447 | chr3 | 64501447 | 3270559 |
|  | F-DMY19.N | 9 | 1 | chr3_65054126 | chr3 | 65054126 | 552679 |
|  | SE21.L | 6 | 1 | chr3_69955293 | chr3 | 69955293 | 4901167 |
|  | ***PH19.N*** | ***6*** | ***7*** | ***chr3_73810222*** | ***chr3*** | ***73810222*** | ***3854929*** |
|  | F-DMY | 7 | 6 | chr3_78458544 | chr3 | 78458544 | 4648322 |
|  | D19.L | 5 | 2 | chr3_80312835 | chr3 | 80312835 | 1854291 |
|  | F-DMY21.L | 2 | 1 | chr3_87521725 | chr3 | 87521725 | 7208890 |
|  | ***F-DMY*** | ***4*** | ***6*** | ***chr3_89061410*** | ***chr3*** | ***89061410*** | ***1539685*** |
|  | PH19.L | 3 | 1 | chr3_89061526 | chr3 | 89061526 | 116 |
|  | ***PH19.N*** | ***2*** | ***7*** | ***chr3_90724627*** | ***chr3*** | ***90724627*** | ***1663101*** |
|  | ***PH19.N*** | ***5*** | ***7*** | ***chr4_5061600*** | ***chr4*** | ***5061600*** |  |
|  | SE19.L | 2 | 1 | chr4_9376996 | chr4 | 9376996 | 4315396 |
|  | PH20.N | 4 | 1 | chr4_11688110 | chr4 | 11688110 | 2311114 |
|  | D19.L | 10 | 2 | chr4_12472542 | chr4 | 12472542 | 784432 |
|  | PH20.N | 5 | 1 | chr4_12533642 | chr4 | 12533642 | 61100 |
|  | PH19.L | 2 | 1 | chr4_16821634 | chr4 | 16821634 | 4287992 |
|  | D19.L | 4 | 2 | chr4_19660246 | chr4 | 19660246 | 2838612 |
|  | FD.L | 5 | 1 | chr4_19906177 | chr4 | 19906177 | 245931 |
|  | SE21.L | 9 | 1 | chr4_27756064 | chr4 | 27756064 | 7849887 |
|  | FD.L | 2 | 1 | chr4_37802113 | chr4 | 37802113 | 10046049 |
|  | SE21.L | 5 | 1 | chr4_50517001 | chr4 | 50517001 | 12714888 |
|  | FD.L | 4 | 1 | chr4_71449166 | chr4 | 71449166 | 20932165 |
|  | F-DMY21.L | 8 | 1 | chr4_75737340 | chr4 | 75737340 | 4288174 |
|  | SE19.L | 4 | 1 | chr5_7337127 | chr5 | 7337127 |  |
|  | FD.L | 3 | 1 | chr5_16136373 | chr5 | 16136373 | 8799246 |
|  | PH19.N | 8 | 7 | chr5_17097132 | chr5 | 17097132 | 960759 |
|  | F-DMY19.L | 7 | 1 | chr5_23802319 | chr5 | 23802319 | 6705187 |
|  | FD.L | 7 | 1 | chr5_60386542 | chr5 | 60386542 | 36584223 |
|  | F-DMY21.L | 7 | 1 | chr5_63752576 | chr5 | 63752576 | 3366034 |
|  | F-DMY19.L | 2 | 1 | chr5_63807728 | chr5 | 63807728 | 55152 |
|  | PH20.L | 8 | 1 | chr5_65695188 | chr5 | 65695188 | 1887460 |
|  | SE19.L | 5 | 1 | chr5_67814609 | chr5 | 67814609 | 2119421 |
|  | F-DMY19.L | 4 | 1 | chr5_71810213 | chr5 | 71810213 | 3995604 |
|  | ***F-DMY20.N*** | ***6*** | ***6*** | ***chr5_72247110*** | ***chr5*** | ***72247110*** | ***436897*** |
|  | PH20.L | 10 | 1 | chr5_79228045 | chr5 | 79228045 | 6980935 |
|  | PH20.L | 3 | 1 | chr6_529239 | chr6 | 529239 |  |
|  | PH20.N | 9 | 1 | chr6_3932011 | chr6 | 3932011 | 3402772 |
|  | FD.L | 6 | 1 | chr6_19510655 | chr6 | 19510655 | 15578644 |
|  | F-DMY21.L | 10 | 1 | chr6_21467039 | chr6 | 21467039 | 1956384 |
|  | F-DMY20.N | 7 | 6 | chr6_32583428 | chr6 | 32583428 | 11116389 |
|  | ***F-DMY20.N*** | ***4*** | ***6*** | ***chr6_40691177*** | ***chr6*** | ***40691177*** | ***8107749*** |
|  | PH21.L | 10 | 1 | chr6_47612620 | chr6 | 47612620 | 6921443 |
|  | PH20.L | 9 | 1 | chr6_64111421 | chr6 | 64111421 | 16498801 |
|  | PH19.L | 6 | 1 | chr6_78800768 | chr6 | 78800768 | 14689347 |
|  | FD.L | 10 | 1 | chr6_79941583 | chr6 | 79941583 | 1140815 |
|  | ***F-DMY20.N*** | ***5*** | ***6*** | ***chr6_82734617*** | ***chr6*** | ***82734617*** | ***2793034*** |
|  | PH19.L | 7 | 1 | chr6_85404793 | chr6 | 85404793 | 2670176 |
|  | SE21.L | 8 | 1 | chr6_87562688 | chr6 | 87562688 | 2157895 |
|  | FD.L | 8 | 1 | chr7_5542653 | chr7 | 5542653 |  |
|  | PH19.N | 4 | 7 | chr7_10758065 | chr7 | 10758065 | 5215412 |
|  | PH19.L | 10 | 1 | chr7_15624550 | chr7 | 15624550 | 4866485 |
|  | F-DMY20.N | 9 | 6 | chr7_30829755 | chr7 | 30829755 | 15205205 |
|  | F-DMY21.L | 4 | 1 | chr7_35249746 | chr7 | 35249746 | 4419991 |
|  | F-DMY19.N | 4 | 1 | chr7_43565881 | chr7 | 43565881 | 8316135 |
| PH21.L | SE21.L | 4-2 | 1-1 | chr7_46045300 | chr7 | 46045300 | 2479419 |
|  | ***F-DMY20.N*** | ***2*** | ***6*** | ***chr7_53172294*** | ***chr7*** | ***53172294*** | ***7126994*** |
|  | PH19.N | 9 | 7 | chr7_62905420 | chr7 | 62905420 | 9733126 |
|  | ***F-DMY20.N*** | ***3*** | ***6*** | ***chr7_62988589*** | ***chr7*** | ***62988589*** | ***83169*** |
|  | SE19.L | 6 | 1 | chr7_65808749 | chr7 | 65808749 | 2820160 |
|  | F-DMY19.N | 9 | 1 | chr7_68142004 | chr7 | 68142004 | 2333255 |
|  | PH20.L | 2 | 1 | chr7_88625236 | chr7 | 88625236 | 20483232 |
|  | ***PH19.N*** | ***3*** | ***7*** | ***chr7_90834458*** | ***chr7*** | ***90834458*** | ***2209222*** |
| PH21.L | SE21.L | 8-7 | 1-1 | chr7_90860527 | chr7 | 90860527 | 26069 |
|  | PH21.L | 7 | 1 | chr8_6019119 | chr8 | 6019119 |  |
|  | F-DMY | 10 | 6 | chr8_12111169 | chr8 | 12111169 | 6092050 |
|  | F-DMY21.L | 3 | 1 | chr8_13069710 | chr8 | 13069710 | 958541 |
|  | PH20.N | 3 | 1 | chr8_13590010 | chr8 | 13590010 | 520300 |
|  | D19.L | 8 | 2 | chr8_14173965 | chr8 | 14173965 | 583955 |
|  | PH19.L | 9 | 1 | chr8_34436529 | chr8 | 34436529 | 20262564 |
|  | SE19.L | 7 | 1 | chr8_35327384 | chr8 | 35327384 | 890855 |
|  | PH19.L | 8 | 1 | chr8_50250536 | chr8 | 50250536 | 14923152 |
|  | PH20.N | 2 | 1 | chr8_51582915 | chr8 | 51582915 | 1332379 |
|  | ***D19.L*** | ***2*** | ***2*** | ***chr8_51582964*** | ***chr8*** | ***51582964*** | ***49*** |
|  | F-DMY21.L | 9 | 1 | chr8_54850132 | chr8 | 54850132 | 3267168 |
|  | SE19.L | 3 | 1 | chr8_69865350 | chr8 | 69865350 | 15015218 |
|  | ***F-DMY*** | ***2*** | ***6*** | ***chr8_74701919*** | ***chr8*** | ***74701919*** | ***4836569*** |

**Supplementary Table 3.** Gene name found under the detected QTLs of our study, and the link to the details.

| Gene | link |
| --- | --- |
| MS.gene010897 | <https://bbric-pipelines.toulouse.inra.fr/myGenomeBrowser/web/iprscan.html?analysis=MSAT_XinJiangDaYe&owner=sebastien.carrere@inrae.fr&accession=MS.gene010897&trackid=57ec684f7a11f99142fa032453dfac92-Msa_protein_interproscan_tsv.tsv> |
| MS.gene014383 | <https://bbric-pipelines.toulouse.inra.fr/myGenomeBrowser/web/iprscan.html?analysis=MSAT_XinJiangDaYe&owner=sebastien.carrere@inrae.fr&accession=MS.gene014383&trackid=57ec684f7a11f99142fa032453dfac92-Msa_protein_interproscan_tsv.tsv> |
| MS.gene020657 | https://bbric-pipelines.toulouse.inra.fr/myGenomeBrowser/web/iprscan.html?analysis=MSAT_XinJiangDaYe&owner=sebastien.carrere@inrae.fr&accession=MS.gene020657&trackid=57ec684f7a11f99142fa032453dfac92-Msa_protein_interproscan_tsv.tsv |
| MS.gene027073 | <https://bbric-pipelines.toulouse.inra.fr/myGenomeBrowser/web/iprscan.html?analysis=MSAT_XinJiangDaYe&owner=sebastien.carrere@inrae.fr&accession=MS.gene027073&trackid=57ec684f7a11f99142fa032453dfac92-Msa_protein_interproscan_tsv.tsv> |
| MS.gene035451 | https://bbric-pipelines.toulouse.inra.fr/myGenomeBrowser/web/iprscan.html?analysis=MSAT_XinJiangDaYe&owner=sebastien.carrere@inrae.fr&accession=MS.gene035451&trackid=57ec684f7a11f99142fa032453dfac92-Msa_protein_interproscan_tsv.tsv |
| MS.gene048728 | <https://bbric-pipelines.toulouse.inra.fr/myGenomeBrowser/web/iprscan.html?analysis=MSAT_XinJiangDaYe&owner=sebastien.carrere@inrae.fr&accession=MS.gene048728&trackid=57ec684f7a11f99142fa032453dfac92-Msa_protein_interproscan_tsv.tsv> |
| MS.gene06565 | [https://bbric-pipelines.toulouse.inra.fr/myGenomeBrowser/web/iprscan.html?analysis=MSAT_XinJiangDaYe&owner=sebastien.carrere@inrae.fr&accession=MS.gene06565&trackid=57ec684f7a11f99142fa032453dfac92-Msa_protein_interproscan_tsv.tsv](https://bbric-pipelines.toulouse.inra.fr/myGenomeBrowser/web/iprscan.html?analysis=MSAT_XinJiangDaYe&owner=sebastien.carrere@inrae.fr&accession=MS.gene75565&trackid=57ec684f7a11f99142fa032453dfac92-Msa_protein_interproscan_tsv.tsv) |
| MS.gene06565 | <https://bbric-pipelines.toulouse.inra.fr/myGenomeBrowser/web/iprscan.html?analysis=MSAT_XinJiangDaYe&owner=sebastien.carrere@inrae.fr&accession=MS.gene06565&trackid=57ec684f7a11f99142fa032453dfac92-Msa_protein_interproscan_tsv.tsv> |
| MS.gene073096 | <https://bbric-pipelines.toulouse.inra.fr/myGenomeBrowser/web/iprscan.html?analysis=MSAT_XinJiangDaYe&owner=sebastien.carrere@inrae.fr&accession=MS.gene073096&trackid=57ec684f7a11f99142fa032453dfac92-Msa_protein_interproscan_tsv.tsv> |
| MS.gene36359 | <https://bbric-pipelines.toulouse.inra.fr/myGenomeBrowser/web/iprscan.html?analysis=MSAT_XinJiangDaYe&owner=sebastien.carrere@inrae.fr&accession=MS.gene36359&trackid=57ec684f7a11f99142fa032453dfac92-Msa_protein_interproscan_tsv.tsv> |
| MS.gene40203 | https://bbric-pipelines.toulouse.inra.fr/myGenomeBrowser/web/iprscan.html?analysis=MSAT_XinJiangDaYe&owner=sebastien.carrere@inrae.fr&accession=MS.gene40203&trackid=57ec684f7a11f99142fa032453dfac92-Msa_protein_interproscan_tsv.tsv |
| MS.gene67894 | https://bbric-pipelines.toulouse.inra.fr/myGenomeBrowser/web/iprscan.html?analysis=MSAT_XinJiangDaYe&owner=sebastien.carrere@inrae.fr&accession=MS.gene67894&trackid=57ec684f7a11f99142fa032453dfac92-Msa_protein_interproscan_tsv.tsv |
| MS.gene98525 | https://bbric-pipelines.toulouse.inra.fr/myGenomeBrowser/web/iprscan.html?analysis=MSAT_XinJiangDaYe&owner=sebastien.carrere@inrae.fr&accession=MS.gene98525&trackid=57ec684f7a11f99142fa032453dfac92-Msa_protein_interproscan_tsv.tsv |
| MsNRG000006g00057221 | <https://medicago.toulouse.inra.fr/MsatMercedes-NRGENE-20181029/?q=MsatMercedes_NRG000006g00057221> |
| MsNRG000037g00224451 | https://medicago.toulouse.inra.fr/MsatMercedes-NRGENE-20181029/?q=MsatMercedes_NRG000037g00224451 |
| MsNRG000105g00468091 | <https://medicago.toulouse.inra.fr/MsatMercedes-NRGENE-20181029/?q=MsatMercedes_NRG000105g00468091> |
| MsNRG000105g00468091 | https://medicago.toulouse.inra.fr/MsatMercedes-NRGENE-20181029/?q=MsatMercedes_NRG000105g00468091 |
| MsNRG000206g00737801 | <https://medicago.toulouse.inra.fr/MsatMercedes-NRGENE-20181029/?q=MsatMercedes_NRG000206g00737801> |
| MsNRG000206g00737911 | <https://medicago.toulouse.inra.fr/MsatMercedes-NRGENE-20181029/?q=MsatMercedes_NRG000206g00737911> |
| MsNRG000350g01027551 | https://medicago.toulouse.inra.fr/MsatMercedes-NRGENE-20181029/?q=MsatMercedes_NRG000003g00035951 |
| MsNRG000350g01027551 | https://medicago.toulouse.inra.fr/MsatMercedes-NRGENE-20181029/?q=MsatMercedes_NRG000350g01027551 |
| MsNRG000410g01125591 | <https://medicago.toulouse.inra.fr/MsatMercedes-NRGENE-20181029/?q=MsatMercedes_NRG000410g01125591> |
| MsNRG000432g01160181 | https://medicago.toulouse.inra.fr/MsatMercedes-NRGENE-20181029/?q=MsatMercedes_NRG000432g01160181 |
| MsNRG000661g01450031 | https://medicago.toulouse.inra.fr/MsatMercedes-NRGENE-20181029/?q=MsatMercedes_NRG000661g01450031 |
| MsNRG000832g01614171 | <https://medicago.toulouse.inra.fr/MsatMercedes-NRGENE-20181029/?q=MsatMercedes_NRG000832g01614171> |
| MsNRG000832g01614181 | <https://medicago.toulouse.inra.fr/MsatMercedes-NRGENE-20181029/?q=MsatMercedes_NRG000832g01614181> |
| MsNRG000840g01620971 | https://medicago.toulouse.inra.fr/MsatMercedes-NRGENE-20181029/?q=MsatMercedes_NRG000840g01620971 |
| MsNRG000921g01687971 | <https://medicago.toulouse.inra.fr/MsatMercedes-NRGENE-20181029/?q=MsatMercedes_NRG000921g01687971> |
| MsNRG000921g01687971 | https://medicago.toulouse.inra.fr/MsatMercedes-NRGENE-20181029/?q=MsatMercedes_NRG000921g01687971 |
| MsNRG001015g01756361 | https://medicago.toulouse.inra.fr/MsatMercedes-NRGENE-20181029/?q=MsatMercedes_NRG001015g01756361 |
| MsNRG001447g01984221 | <https://medicago.toulouse.inra.fr/MsatMercedes-NRGENE-20181029/?q=MsatMercedes_NRG001447g01984221> |
| MsNRG001933g02106001 | <https://medicago.toulouse.inra.fr/MsatMercedes-NRGENE-20181029/?q=MsatMercedes_NRG001933g02106001> |
| MsNRG066413g02467211 | https://medicago.toulouse.inra.fr/MsatMercedes-NRGENE-20181029/?q=MsatMercedes_NRG066413g02467211 |
| MsNRG143424g02636541 | https://medicago.toulouse.inra.fr/MsatMercedes-NRGENE-20181029/?q=MsatMercedes_NRG143424g02636541 |
| MtrunA17Chr2g0320811 | <https://medicago.toulouse.inra.fr/MtrunA17r5.0-ANR/?q=MtrunA17_Chr2g0320811> |
| MtrunA17Chr2g0331051 | <https://medicago.toulouse.inra.fr/MtrunA17r5.0-ANR/?q=MtrunA17_Chr2g0331051> |
| MtrunA17Chr2g0331061 | <https://medicago.toulouse.inra.fr/MtrunA17r5.0-ANR/?q=MtrunA17_Chr2g0331061> |
| MtrunA17Chr3g0128361 | <https://medicago.toulouse.inra.fr/MtrunA17r5.0-ANR/?q=MtrunA17_Chr3g0128361> |
| MtrunA17Chr3g0128361 | https://medicago.toulouse.inra.fr/MtrunA17r5.0-ANR/?q=MtrunA17_Chr3g0128361 |
| MtrunA17Chr3g0128381 | https://medicago.toulouse.inra.fr/MtrunA17r5.0-ANR/?q=MtrunA17_Chr3g0128381 |
| MtrunA17Chr3g0128381 | https://medicago.toulouse.inra.fr/MtrunA17r5.0-ANR/?q=MtrunA17_Chr3g0128381 |
| MtrunA17Chr3g0143411 | https://medicago.toulouse.inra.fr/MtrunA17r5.0-ANR/?q=MtrunA17_Chr3g0143411 |
| MtrunA17Chr3g0143411 | https://medicago.toulouse.inra.fr/MtrunA17r5.0-ANR/?q=MtrunA17_Chr3g0143411 |
| MtrunA17Chr7g0228011 | https://medicago.toulouse.inra.fr/MtrunA17r5.0-ANR/?q=MtrunA17_Chr7g0228011 |
| MtrunA17Chr7g0240251 | https://medicago.toulouse.inra.fr/MtrunA17r5.0-ANR/?q=MtrunA17_Chr7g0240251 |
| MtrunA17Chr7g0240251 | https://medicago.toulouse.inra.fr/MtrunA17r5.0-ANR/?q=MtrunA17_Chr7g0240251 |
